# Analysis of Gut Microbiota and Their Metabolic Potential in Patients with Schizophrenia Treated with Olanzapine: Results from a Six-Week Observational Prospective Cohort Study

**DOI:** 10.3390/jcm8101605

**Published:** 2019-10-03

**Authors:** Justyna Pełka-Wysiecka, Mariusz Kaczmarczyk, Agata Bąba-Kubiś, Paweł Liśkiewicz, Michał Wroński, Karolina Skonieczna-Żydecka, Wojciech Marlicz, Błażej Misiak, Teresa Starzyńska, Jolanta Kucharska-Mazur, Igor Łoniewski, Jerzy Samochowiec

**Affiliations:** 1 Department of Psychiatry, Pomeranian Medical University in Szczecin, Broniewskiego 26, 71-460 Szczecin, Poland; wysiecki@wp.pl (J.P.-W.); agata0621@gmail.com (A.B.-K.); pjliskiewicz@gmail.com (P.L.); mwronski@pum.edu.pl (M.W.); jola_kucharska@tlen.pl (J.K.-M.); samoj@pum.edu.pl (J.S.); 2 Department of Clinical and Molecular Biochemistry, Pomeranian Medical University in Szczecin, Powstańców Wielkopolskich 72, 70-111 Szczecin, Poland; mariush@pum.edu.pl; 3 Department of Human Nutrition and Metabolomics, Pomeranian Medical University in Szczecin, Broniewskiego 24, 71-460 Szczecin, Poland; karzyd@pum.edu.pl; 4 Department of Gastroenterology, Pomeranian Medical University in Szczecin, Unii Lubelskiej 1, 71-252 Szczecin, Poland; marlicz@hotmail.com (W.M.); testa@pum.edu.pl (T.S.); 5 Department of Genetics, Wroclaw Medical University, Marcinkowskiego 1, 50-368 Wrocław, Poland; mblazej@interia.eu

**Keywords:** microbiota, schizophrenia, olanzapine administration, weight gain

## Abstract

Accumulating evidence indicates the potential effect of microbiota on the pathogenesis and course of schizophrenia. However, the effects of olanzapine, second-generation antipsychotics, on gut microbiota have not been investigated in humans. This study aimed to analyze fecal microbiota in schizophrenia patients treated with olanzapine during six weeks of their hospital stay. After a seven-day washout from all psychotropic medications, microbiota compositions were evaluated at baseline and after six weeks of hospitalization using 16S rRNA sequencing. The study was conducted in 20 inpatients, who followed the same hospital routine and received 5–20 mg daily doses of olanzapine. Olanzapine treatment was associated with clinical improvements in all patients and significant increases in body mass index in females, but not changes in gut microbiota compositions and predicted function. The severity of symptoms at the beginning of treatment varied in accordance with the predicted metabolic activity of the bacteria. The present findings indicate that the microbiota of schizophrenia patients is highly individual and has different taxonomical (Type 1, with a predominance of *Prevotella*, and Type 2 with a higher abundance of *Bacteroides*, *Blautia* and *Clostridium*) and functional clusters, and it does not change following six weeks of olanzapine therapy; in addition, the microbiota is not associated with either the weight gain observed in women or the effectiveness of olanzapine therapy.

## 1. Introduction

More than 21 million people worldwide suffer from schizophrenia (SZ) [1]. A growing body of studies has shown the role of the gut–brain axis dysregulation in the pathophysiology of SZ. Subclinical inflammation, aberrant monoamine metabolism, and abnormal hypothalamic–pituitary–adrenal axis activation have been widely reported in patients with SZ [2,3,4,5] and are associated with microbiota alterations [6,7,8,9]. For instance, Schwartz et al. [10] found elevated abundance of Lactobacillaceae, Halothiobacillaceae, Brucellaceae, and Micrococcineae and lowered counts of Veillonellaceae in a cohort of SZ patients; in addition, greater microbial abnormalities, lower remission rates, and poorer responses to therapy, as well as decreased microbiome α-diversity index and altered gut microbial composition, were observed in SZ patients [11]. Although mechanisms underlying the potential effect of microbiota on the pathogenesis and course of SZ are yet to be determined, chronic inflammation [12] and altered tryptophan metabolism [13,14] have been suggested to be implicated in the pathogenesis of SZ. However, gut microbiota-associated biomarkers that would hold clinical utility have not been indicated to date. 

Olanzapine (OLZ), one of the most widely used second-generation antipsychotics (SGAs) [15], has multiple adverse effects, including weight gain, dyslipidemia, impaired glucose metabolism, and hypertension [16,17,18,19]. These metabolic adversities may occur shortly after treatment implementation and progress with treatment duration [20,21,22]. Importantly, the first year of antipsychotic treatment is a critical period for weight gain and other metabolic adverse effects [23]. Notably, weight gain at the beginning of OLZ therapy can be used to predict long-term outcomes related to cardiovascular comorbidity. Therefore, dietary counseling and weight management, including regular bodyweight measurements, should be implemented as soon as the OLZ therapy begins [24,25]. However, weight gain is of multifactorial nature [20,26,27,28], and, to date, no effective therapeutic strategies could prevent weight gain in patients treated with OLZ.

A few studies have demonstrated that OLZ administration plays a role in weight gain and metabolic malfunctions. Davey et al. [29] found that OLZ treatment induced metabolic alterations via microbiota changes, and the metabolic alterations could be reversed by treatment with antibiotics; in addition, microbial, inflammatory, and metabolic adversities related to OLZ treatment were sex-dependent [30]. Moreover, Morgan et al. [31] observed that weight gain depended on gut microbiota, and specific bacteria were responsible for weight gain. Furthermore, Flowers et al. [32] revealed that clusters of gut microbiota were associated with pharmacological treatment in patients with bipolar disorder. However, to the best of our knowledge, the effects of OLZ on gut microbiota in patients with SZ have not been investigated. We hypothesized that short-term treatment with OLZ in controlled conditions (unified dietary intake and environmental factors) affects fecal microbiota compositions, and microbiota can affect body weight and treatment efficacy. Accordingly, this study analyzed microbiota compositions of stool samples collected from a cohort of SZ inpatients. The cohort comprised of acutely-relapsed SZ inpatients who were followed-up for six weeks during OLZ treatment. 

## 2. Materials and Methods

### 2.1. Patients

**Figure 1:**
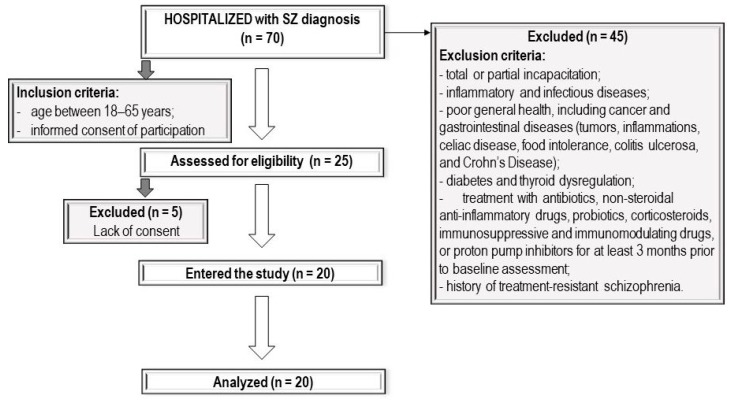
Flow chart of the study design. SZ, schizophrenia.

The study protocol was approved by the Bioethics Committee of the Pomeranian Medical University in Szczecin (Poland). All participants received a written description of the study aims and provided written informed consent before participation. Participants were recruited as inpatients at the Department of Psychiatry in Szczecin (Poland) between October 2016 and May 2018, and only 20 psychiatric inpatients met the inclusion criteria. The flow chart of the study design is shown in Figure 1. SZ was diagnosed based on the ICD (International Statistical Classification of Diseases and Related Health Problems) −10 criteria. 

### 2.2. Study Protocol

All participants were subjected to the same daily activities, including physical exercise (daily morning exercise and a walk with a therapist), occupational therapy, and psycho-educational activities. Two senior psychiatrists performed the psychiatric and basic physical examinations, and a gastroenterologist conducted a comprehensive physical examination.

Patients received a standard hospital diet (i.e., 2995 ± 93 kcal, 106 ± 14 g total protein, 420 ± 24 g carbohydrates, and 102 ± 10 g fat per day), balanced by a hospital dietician, in accordance with the Polish standards for hospitalized patients [33]. Detailed nutritional data on the diet during hospitalization, including fiber consumption, are presented in Supplementary Table S1.

This study included 20 patients, with 11 males and 9 females. After admission to the hospital ward, they were all subjected to a 7-day washout from psychiatric medications, received the standard hospital diet, and had a similar hospital routine. The first stool samples were collected after the washout period (W0), and subsequently, OLZ treatment was administered (initially 5 mg/day; doses were individually adjusted up to 20 mg/day). After 6 weeks of treatment, the second stool samples (W6) were collected (Supplementary Figure S1).

Clinical responses were defined as follows: Early responders, 30% reduction in positive and negative syndrome scale (PANNS) total score at 4 weeks; late responders, 40% reduction in PANNS total score at end-point [34]; Clinical global impression-improvement scale (CGI-I) responders, score of 3 points (much improvement); and non-responders, clinical global impression-severity (CGI-S) scores of 4 (minimal improvement) or 5 (no improvement).

### 2.3. Processing of Raw Data and Statistical Analysis

Sequencing of the V4 region of 16S rRNA gene was performed by the uBiome, Inc. (San Francisco, CA, USA). The 16S amplicons from each sample were individually barcoded and sequenced in the multiplex in the NextSeq 500 platform in a 150 bp (base pair) paired-end modality. The initial quality check of the 16S sequences was conducted using the AfterQC (version 0.9.7) software with default settings [35]. Subsequently, forward and reverse reads were, respectively, capped at 125 and 124 bp and then joined together with an in-between padding sequence (8 of “Ns” with a base score quality of 40). Each sequence was assigned the number of expected errors, and the sequences were filtered to have a maximum expected error of 1.0. The above steps were conducted using the VSEARCH (2.8.0) tool [36]. The sequences were processed using mothur (v.1.41.3) [37]. Briefly, sequences were aligned to the SILVA bacterial reference alignment (release 132), and were then screened to drop those not aligning to positions 13,148 and 25,277 of the SILVA alignment and were pre-clustered to allow two differences between sequences. The chimeras were identified and removed using VSEARCH implemented in mothur. Subsequently, sequences were classified using a Wang method with the Greengenes 16S rRNA Database version 13.8. Finally, sequences were clustered into OTUs using opticlust algorithm and Matthews correlation coefficient metric. 

Metagenomic predictions from 16S rRNA marker genes (corrected for predicted 16S rRNA copy number) were carried out using PICRUSt (version 1.1.3) [38], and a list of the KEGG (Kyoto Encyclopedia of Genes and Genomes) functional orthologs (KO) was created. Reference genome coverage of the samples was calculated using the weighted nearest sequenced taxon index (NSTI) [38]. The PICRUSt predicted a median NSTI score of 0.11 (interquartile range, IQR of 0.05). The predicted metagenomes were analyzed with HUMAnN [39] and LEfSe [40]. The KO list was submitted as input data to HUMAnN, which generated KEGG modules and KEGG pathway abundances.

Downstream data analysis was performed using the R software (version 3.5.1, https://cran.r-project.org/), R based tools (such as Phyloseq package (version 1.24.2)) [41] and ComplexHeatmap [42], and custom-made scripts. Before calculating alpha diversity, the samples were rarefied to 3680 sequences per sample. Prior to beta diversity analysis, the taxa with the prevalence of less than 5% were removed (the prevalence of taxa was defined as the proportion of samples in which the taxa appeared at least once). Beta diversity was analyzed using principal coordinate analysis (PCoA) on Bray–Curtis distance matrices generated from the relative OTU abundances. To analyze the changes in bacterial community composition, a change in the principal coordinate 1 (PC1) was examined. The statistical analysis methods included the Wilcoxon rank-sum test, paired Wilcoxon signed-rank test, *t*-test for one sample, and Spearman rho correlation coefficient. *p*-values were adjusted using the Benjamini–Hochberg’s false discovery rate (FDR) controlling procedure. Numerical data are presented as median, lower quartile, and upper quartile. 

## 3. Results

### 3.1. Microbiota Compositions

**Figure 2:**
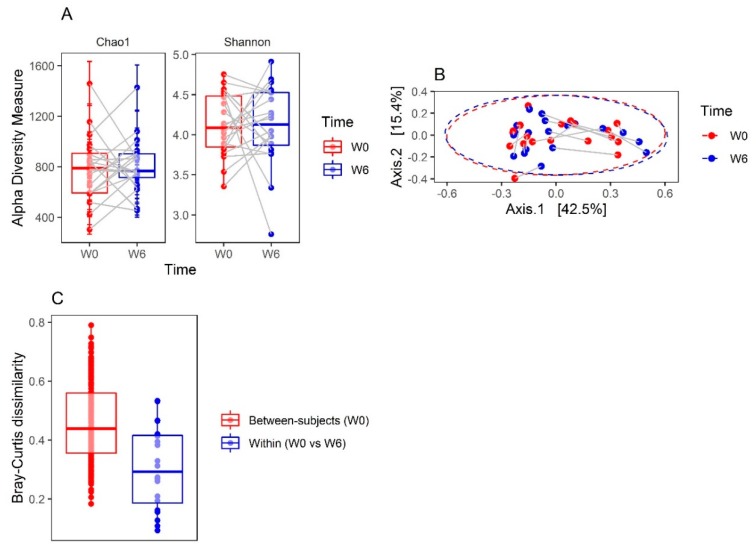
(**A**) Alpha diversity measures at baseline (W0) and after six weeks of hospitalization (W6). The boxplots represent the diversity measures (center line, median; lower and upper hinges correspond to the first (Q1) and third (Q3) quartiles; whiskers, 1.5 * IQR (Q3–Q1). Grey lines connect samples from the same patients. (**B**) Genus level resolution analysis of gut microbiota in patients diagnosed with paranoid schizophrenia treated with olanzapine during six weeks of hospitalization. The principal coordinate analysis was based on Bray–Curtis dissimilarities calculated using relative abundance data. Samples are colored according to time points (W0 and W6). Grey lines connect samples from the same patients. Ellipses correspond to 95% confidence intervals for two timepoints (W0 and W6) with a multivariate normal distribution. (**C**) The boxplot shows Bray–Curtis dissimilarities calculated in the same patients (within (W0 vs. W6), 0.29 (0.19–0.42)) and in different patients (between subjects (W0), 0.44 (0.36–0.56), *p* = 0.00006, Wilcoxon rank-sum test) (center line: median, lower, and upper hinges correspond to the first (Q1) and third (Q3) quartiles; whiskers: the upper whisker is located at the smaller of the maximum Bray–Curtis measures and Q3 + 1.5 * IQR (Q3–Q1); the lower whisker is located at the larger of the minimum Bray–Curtis measures and Q1—1.5 * IQR). W0 and W6 represent time points.

General characteristics of patients are shown in Table 1. There was no significant change in alpha diversity as measured by Chao1 and Shannon indexes (*p* = 0.955 and *p* = 0.808, respectively; Figure 2A). The PCoA with Bray–Curtis dissimilarity is presented in Figure 2B. Samples were separated into distinct regions, mainly along the PC1 (Axis.1) that explained 42.5% of the intersample variance. The gut microbiome was individually specific, and the Bray–Curtis distances between the same samples were significantly smaller than those between all W0 samples (*p* = 0.00006; Figure 2C). The direction of change along the PC1 was not consistent (Supplementary Figure S2). The mean change in the PC1 was not significantly different from 0 (0.0012, (95% confidence interval: −0.0946, 0.0970), *t* = −0.03, df = 19, *p* = 0.979), suggesting that the gut microbial community composition does not change after six weeks of treatment. In line with this observation, no OTUs were differentially abundant (from the genus to phylum level) between W0 and W6 (Supplementary Figures S3–S5). There was no change in the ratio of Firmicutes to Bacteroidetes (F/B) in the whole group, as well as in males and females (Supplementary Figure S6). In addition, there were no significant differences in the abundance of the KEGG orthologs, modules, and pathways between W0 and W6 samples in the whole group, as well as in men and women (Supplementary Figure S7).

**Figure 3:**
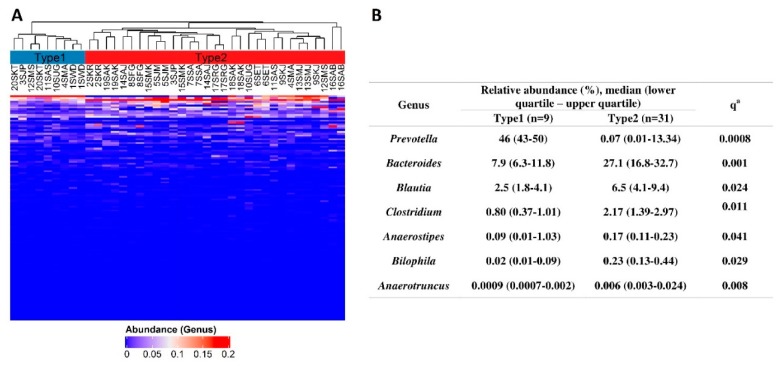
(**A**) Genus level resolution analysis of gut microbiota in patients diagnosed with paranoid schizophrenia treated with olanzapine during six weeks of hospitalization. Unsupervised average linkage hierarchical clustering of gut microbiota at the genus level was conducted. Two resulting clusters (Type 1, blue and Type 2, red) are shown as the top annotation. Both samples (W0 and W6) of 15 patients were found in either Type 1 or Type 2 cluster (two patients in Type 1 and 13 patients in Type 2). Samples of the five patients (3SJP, 4SMA, 10SUG, 11SAS, and 12SMS) belonged to different clusters. (**B**) Differential abundance testing at the genus level between Type 1 and Type 2 clusters. ^a^ two sided Wilcoxon signed-rank test, FDR adjusted p, the genera with the relative abundance >1% in at least one cluster are shown, *Eggerthella* not shown due to low abundance.

Despite the lack of a consistent shift along the PC1, we examined whether the PC1 changes are associated with demographic, clinical, and environmental factors. The mean PC1 changes did not differ between men (0.011 (−0.118–0.140)) and women (0.004 (−0.036–0.016)) (Wilcoxon rank-sum test FDR adjusted *p* (*q*) = 0.649). Demographic, clinical, and environmental factors were not correlated with the change in the PC1 (Supplementary Table S2, Supplementary Figure S8). There was no association between the dose of OLZ and the shift in the gut microbial composition (Supplementary Figure S9). However, the change in the PC1 was significantly greater in patients consuming alcohol (1–3 unit of alcohol; 0.16 (0.03–0.32)) than in non-alcohol drinkers (−0.01 (−0.19–0.01)) (Wilcoxon rank-sum test *q* = 0.036). To further explore the distinct regions revealed by the ordination of samples by PCoA (Figure 2B), we conducted an unsupervised hierarchical clustering using an average linkage algorithm of the Bray–Curtis dissimilarity. The clustering analysis showed the presence of two clusters (Type 1: 9 samples; Type 2: 31 samples) that matched with distinct regions revealed in the PCoA. A heatmap displaying the relative abundances for the genera annotated with two resulting clusters is shown in Figure 3A. Differential abundance testing revealed that the *Bacteroides*, *Blautia*, *Clostridium*, *Anaerostipes*, *Bilophila*, *Anaerotruncus,* and *Eggerthella* were enriched in the Type 2 cluster, whereas *Prevotella* was enriched in the Type1 cluster (Figure 3B). Clusters Type 1 and 2 seemed to correspond to enterotypes 2 (*Prevotella*) and 1 (*Bacteroides*) described by Arumugam et al. in 2011 [43], respectively. To explore these enterotypes in more detail, our cluster Type 2 was analyzed more thoroughly, as it seemed not to be completely homogenous. Cluster Type 2 was divided into Type 2A and Type 2B, and then the relative abundances of the main contributors from each enterotype in the resulting three clusters (Type 1, Type 2A and 2B) were ascertained (Supplementary Figure S10) Cluster Type 1 had an abundance pattern similar to enterotype 2 (Figure 2d in Arumugam et al. [43]), and that for cluster type 2A to enterotype 1 (Figure 2d in Arumugam et al. [43]). However, cluster type 2B seemed not to be similar to any enterotype. Two genera (*Prevotella* and *Bacteroides*) exhibited similar abundance which was greater than of *Ruminococcus*. The pattern Type 2B seemed to be a type of a mixture from clusters Type 1 and Type 2A. The addition of *Blautia* made no difference to this assessment (Supplementary Figure S11). 

Taken together, our results suggest that the gut microbiota is highly individually specific, and the microbial community compositional changes during six weeks of OLZ treatment are not consistent across the patients. 

### 3.2. Clinical Improvement and BMI Changes

We found that OLZ treatment was associated with significantly improved treatment efficacy as measured by PANNS, 36-item short form survey (SF36), and CGI-S scales (Supplementary Table S3). We further investigated whether these improvements are correlated with the change in microbiota compositions (as measured by a change in the PC1 component) and with demographic and clinical characteristics. No significant correlations were observed between clinical improvements and changes in microbiota composition (Supplementary Figure S12) or demographic and clinical characteristics, except the duration of untreated psychosis (DUP) (Supplementary Table S4). 

In contrast to changes in the symptom severity of schizophrenia (Supplementary Table S3), there was no significant change in the patients’ BMI during OLZ treatment (*q* = 0.763). However, the BMI change (W6 vs. W0 difference) was significantly higher in women than in men (Supplementary Figure S13) but did not correlate significantly with age, OLZ average dose per day, OLZ maximum dose, disease duration, or duration of untreated psychosis.

**Figure 4:**
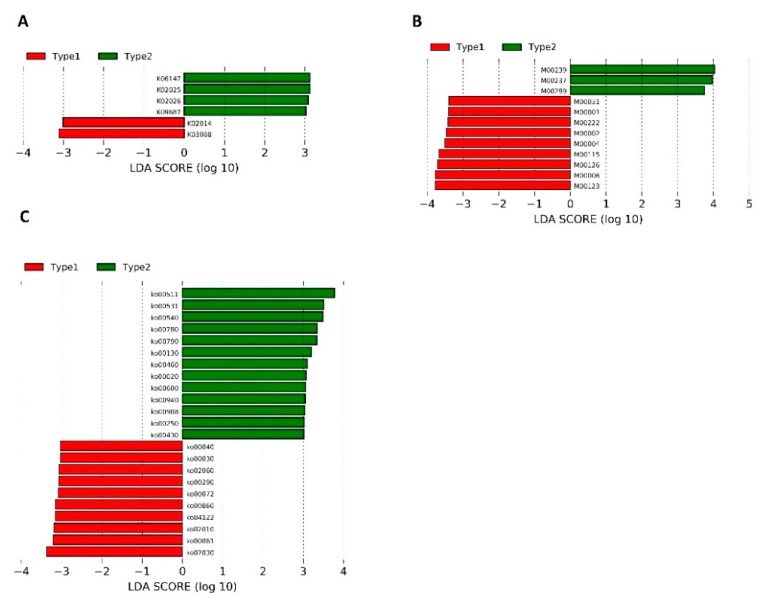
Unsupervised average linkage hierarchical clustering was carried out for each KEGG feature category. (**A**) KEGG orthologs; (**B**) KEGG modules; (**C**) KEGG pathways. K06147, ATP-binding cassette, subfamily B, bacterial; K02025, K02026, multiple sugar transport system permease proteins; K09687, antibiotic transport system ATP-binding protein; K02014, iron complex outer-membrane receptor protein; K03088, RNA polymerase sigma-70 factor, ECF subfamily; M00239, peptides/nickel transport system; M00237, branched-chain amino acid transport system; M00299, Spermidine/putrescine transport system; M00051, Uridine monophosphate biosynthesis, glutamine (+ PRPP) → UMP; M00222, phosphate transport system; M00002, glycolysis, core module involving three-carbon compounds; M00004, pentose phosphate pathway (pentose phosphate cycle); M00115, NAD biosynthesis, aspartate → NAD; M00126, Tetrahydrofolate biosynthesis, GTP → THF; M00006, pentose phosphate pathway, oxidative phase, glucose 6P → ribulose 5P; M00123, Biotin biosynthesis, pimeloyl-CoA → biotin; ko00511, other glycan degradation; ko00531, glycosaminoglycan degradation; ko00540, lipopolysaccharide biosynthesis; ko00780, biotin metabolism; ko00790, folate biosynthesis; ko00130, ubiquinone and other terpenoid–quinone biosynthesis; ko00460, cyanoamino acid metabolism; ko00020, citrate cycle (TCA cycle); ko00600, sphingolipid metabolism; ko00940, phenylpropanoid biosynthesis; ko00908, zeatin biosynthesis; ko00250, alanine, aspartate and glutamate metabolism; ko00430, taurine and hypotaurine metabolism; ko00040, pentose and glucuronate interconversions; ko00030, pentose phosphate pathway; ko02060, phosphotransferase system (PTS); ko00290, valine, leucine and isoleucine biosynthesis; ko00072, synthesis and degradation of ketone bodies; ko00860, porphyrin and chlorophyll metabolism; ko04122, sulfur relay system; ko02010, ABC transporters; ko00061, fatty acid biosynthesis; ko02030, bacterial chemotaxis.

Because we found clear differences in gut microbiome compositions in all 40 samples (Figure 3), we next sought to determine whether similar differences in microbial community compositions and metabolic potentials exist in baseline samples and whether those differences could affect the patients’ clinical improvement and change in BMI within six weeks. We performed the unsupervised average linkage hierarchical clustering of the Bray–Curtis dissimilarity among the baseline samples (W0, Supplementary Figure S14), as well as that of the relative abundances of the predicted KEGG orthologs, modules, and pathways (Supplementary Figures S15–S17). Regarding the microbiome compositions, we were able to demonstrate different groups of patients (clusters) using hierarchical clustering of KEGG features in the W0 samples: KEGG orthologs (Supplementary Figure S15), modules (Supplementary Figure S16), and pathways (Supplementary Figure S17). Differential abundance testing revealed that only the *Prevotella* genus differed between the two clusters (Type 1, 0.01% (0.006–0.004) vs. Type 2, 27.4% (17.7–43.1); two-sided Wilcoxon signed-rank test, FDR adjusted *p* = 0.033; Supplementary Figure S14). To identify differentially abundant genes, modules, and pathways between clusters, we conducted a linear discriminant analysis with effect size (LEfSe) method (Figure 4). 

Subsequently, we compared the baseline symptom scales and BMI between Type 1 and Type 2 clusters. We found significant differences in the baseline PANNS, PANNS G, and CGI-S between the groups created from the clustering of the pathway abundance (Table 2). The patients classified into a Type 2 cluster had significantly more severe symptoms at baseline. The improvement in symptom severity after OLZ treatment assessed by PANNS, SF36, and CG1I was not associated with microbial community compositions (Supplementary Figure S14, Table S5) or KEGG features at baseline (Table 2; Supplementary Figures S15–S17 and Tables S6 and S7). Likewise, no associations were found between baseline gut microbiota (Supplementary Figure S14, Supplementary Table S5) or its metabolic potentials (Table 2 and Supplementary Figures S15–S17 and Supplementary Tables S6 and S7) and the BMI change in the whole group or separately in women or men.

To further explore the gut microbiota and OLZ treatment interactions, we classified the included patients as responders and non-responders as follows: Early responders, early non-responders, late responders, and late non-responders using the PANNS total score and responders and non-responders using the CGI-I scale. Subsequently, microbial community compositions and KEGG features were compared between responders and non-responders. Phylogenetic compositions of the samples at the phylum level in the responders and non-responders are shown in Supplementary Figure S18. The phyla were not differentially abundant in responders and non-responders, regardless of the definition of clinical improvement. There were no differences in gut microbiome compositions at other taxonomic levels (Supplementary Figure S19), as well as in the KEGG orthologs, modules, and pathways (Supplementary Figure S20). Sex-specific results are shown in Supplementary Figures S21 and S22 (bacterial community composition) and S23 and S24 (KEGG features).

## 4. Discussion

The effect of OLZ on the microbiota has been investigated in experimental studies. Davey et al. [29,30] found decreased gut microbiota diversity, increased abundance of phyla Firmicutes, and reduced Actinobacteria, Proteobacteria, and Bacteroidetes in the course of OLZ treatment in female rats. Similarly, Morgan et al. [31] revealed decreased alpha diversity, lower abundance of class Bacteroidia, and increased abundances of Erysipelotrichia, Actinobacteria, and Gammaproteobacteria in female mice treated with OLZ. However, Kao et al. [44] demonstrated no significant effects of OLZ on gut microbiota in female rats. To the best of our knowledge, this study is the first to analyze fecal microbiota compositions in patients hospitalized due to acute relapse of SZ. We did not find the impact of six-week OLZ treatment on bacterial diversity, abundance, and predicted metabolic function, and patients with SZ had individualized and stable gut microbiota in the course of six-week OLZ treatment in terms of both composition and function. Because of the inconsistent findings above, further studies are needed to clarify the effect of OLZ on gut microbiota.

Although gut microbiota could be compositionally and functionally clustered into similar groups, the classification could not be used to predict the responses to OLZ treatment or the occurrence of weight gain (observed only in women) during OLZ treatment. As mentioned above, OLZ causes weight gain in female rats [29,44] and mice [31]. This metabolic effect is not observed during antibiotic therapy [29] and gnotobiosis (germ-free mouse model) and is enhanced during the administration of the high-fat diet regimen that is responsible for alterations of microbiota similar to those observed in metabolic syndromes [31]. In addition, Davey et al. [30] demonstrated metabolic disturbances, inflammation, and microbiota alterations in female mice treated with OLZ and found only slight alterations in male mice treated with OLZ, and metabolic effects of OLZ were linked to gut microbiota alterations. Notably, antibiotics reversed these effects due to reduced gut microbiota. Therefore, changed gut microbiota plays a pivotal role in weight gain. The lack of association between fecal microbiota compositions and weight gain in this study may be due to the low number of participants and the short period of OLZ administration. In addition, other factors might also be responsible for the increase in body mass index associated with the OLZ administration [20,26,27,28].

In the present study two bacterial enterotypes (clusters) were found, Type 1, with a predominance of *Prevotella*, and Type 2 with a higher abundance of *Bacteroides*, *Blautia* and *Clostridium*. Cluster Type 2 seemed not to be completely homogenous (with Types 2A and 2B), which initially suggested the possibility of the occurrence of a third enterotype similar to that found by Arumugam et al. [43]. Further analysis did not confirm this hypothesis and a higher abundance of *Ruminococcus* or *Blautia* in sub-cluster Type 2B was not seen. This sub-cluster seemed to be a type of mixture from clusters Type 1 and Type 2A. Due to this we took into consideration in further analyses only two enterotypes (original clusters) of bacteria. Moreover, patients with SZ were clustered at the level of KEGG genes, modules, and pathways. The severity of symptoms measured at the beginning of treatment varied, depending on the predicted metabolic activity of the bacteria. Other studies also have observed a relationship between the composition of bacteria and the severity of symptoms in SZ patients. Zheng et al. [11] demonstrated that PANSS was negatively correlated with Veillonellaceae and was positively correlated with Bacteroidaceae, Streptococcaceae, and Lachnospiraceae. Schwartz et al. [10] found greater microbial abnormalities in SZ patients than in controls. In addition, increases in the number of Lactobacillus group bacteria were positively correlated with the severity of various symptom domains in SZ patients and were negatively correlated with the global assessment of functioning. Moreover, responses to the treatment were worse in patients with severe microbiota alterations. Furthermore, Shen et al. [45], using the PICRUSt analysis, infer that vitamin B6 and fatty acid metabolic potential differed significantly between SZ patients and controls. Therefore, there are potential relationships between predicted metabolic changes and the severity of symptoms in SZ patients, as shown in Table 3. It is important to note that the PICRUSt approach using in prediction of bacterial metabolic activity should be treated with caution and followed by metagenomic analyses to explain such findings in humans. The median NSTI score was 0.11 (interquartile range of 0.05) suggesting a reasonable accuracy of the prediction, however, some closely related reference genomes were not available.

Our study has several strengths that should be highlighted. (1) The applied treatment resulted in expected clinical effects. The relationship between duration of untreated psychosis (DUP) and poor general symptomatic outcomes was confirmed, and the longer DUP was associated with more severe positive and negative symptoms. Additionally, OLZ treatment caused weight gain. This observation is in agreement with that in another experimental study [65]. (2) During the treatment, the patients were under the same controlled hospital conditions (diet, drug intake, and clinical monitoring), and a washout was used before treatment, thus providing a "unification" of the environmental impact on the fecal microbiota pattern. Consequently, we speculate that such conditions diminish the impact of common environmental factors that permanently shape gut microbiota composition and underline the association between the disease and treatment. (3) Weight gain at the beginning of OLZ treatment is very important because it determines the further development of cardiometabolic risk factors [22,23,66]. (4) Although the study group was not homogeneous, the symptoms were observed every day in our psychiatric clinic. Patients were previously treated with other pharmaceuticals, which might have affected the microbiota composition. Such situations might lead to resistance against psychotropic drugs, probably leading to no impact of OLZ on the microbiota.

There are certain limitations of our study that need to be discussed. First, the sample size was small and heterogeneous (drug-naive and previously-treated patients). No formal sample size calculations were employed for this analysis, but the cohort size was based on what was previously sufficient to test microbiotic changes in schizophrenia patients [67], and/or the influence of antibiotics [68] and risperidone administration [69] on gut microbiota. This limitation should be attributed to rigorous inclusion and exclusion criteria as well as the short duration of the whole study (17 months). Thus, studies with a greater sample size are needed to further examine the associations between OLZ treatment and gut microbiota structure. Second, the composition of intestinal bacteria varied among individuals, and inter-individual variation within the gut ecosystem of patients was high. Third, in individual studies (also experimental), various taxonomic groups of bacteria were analyzed only in stools. The composition of bacteria in feces is more stable and is not influenced by external factors compared with the composition of bacteria in the small intestine. Changes in the microbiota of the small bowel have a much greater effect on the metabolic functions of the human body. Therefore, further experimental studies should pay more attention to this issue [70,71], although an invasive way of sampling intestine biological material remains difficult and holds several ethical concerns. Fourth, there was a lack of long-term follow-up, which is especially important in case of metabolic consequences of OLZ treatment. Fifth, we did not compare the results between SZ patients and healthy subjects or patients receiving placebos. Matched controls with similar lifestyle should be used to exclude false-positive results. However, the general lifestyle in patients diagnosed with SZ was found to be divergent from that observed in healthy people [72]. Therefore, a placebo approach was impossible mainly due to ethical and organizational concerns. Sixth, changes in dietary and living conditions during the hospital stay might be another limitation of our study. However, enterotypes *Prevotella* and *Bacteroides* are strongly associated with long-term diet. It was shown that microbiome composition changed detectably within 24 h of initiating a high-fat/low-fiber or low-fat/high-fiber diet, but that enterotype identity remained stable during the 10-day study [73]. Therefore, a change of diet after admission to hospital should not affect W0 microbiota. After this all patients received the same diet, and it seems that this factor should also not significantly affect the influence of OLZ on W6 microbiota composition.

## 5. Conclusions

In conclusion, the present findings indicate that the microbiota in patients with the schizophrenia episode is highly individualized, although it can be clustered into different taxonomical (Type 1, with a predominance of *Prevotella*, and Type 2 with a higher abundance of *Bacteroides*, *Blautia,* and *Clostridium*) and functional groups; the microbiota does not change during six weeks of treatment with OLZ and is not associated with the weight gain that occurs in women treated with OLZ, as well as the treatment effectiveness. This study provides some insights into the metabolic effects of psychotropic drugs on gut microbiota in SZ patients. Further long-term and placebo-controlled studies are needed to clarify the effect of OLZ on gut microbiota.

## Figures and Tables

**Table 1:** Clinical characteristics of patients included in the study (*n* = 20).

Variables	Median (1st Q–3rd Q)/ *n* (%)
Sex (F/M)	9 (45%)/11 (55%)
Age (years)	33.5 (31–39)
BMI (kg/m^2^)	28.91 (24.82–31.27)
Olanzapine maximum dose (mg)	20.00 (20.0–20.0)
Olanzapine average dose per day (mg)	15.54 (13.50–16.34)
Disease duration (months)	90 (32–114)
Duration of untreated psychosis (months)	4.5 (1.75–12.0)
Smoking (number of cigarettes per day) ^a^	1.5 (1.0–3.0)
Coffee (number of cups)	2.0 (0.0–3.0)
Tea (number of cups)	2.5 (1.0–3.0)

BMI—Body Mass Index; ^a^ Ordinal variables (per day): 1, non-smokers; 2, up to 10 cigarettes; 3, up to 20 cigarettes; 4, up to 40 cigarettes; 1st Q, first quartile; 3rd Q, third quartile, BMI—body mass index.

**Table 2:** Associations of KEGG pathways with BMI changes and clinical improvements (PANNS, SF36, and CGI).

**Variables (Females + Males)**	**Cluster Type 2 (*n* = 5)**	**Cluster Type 3 (*n* = 12)**	***p*/*q*^a^**
BMI (kg/m^2^) W0	28.7 (27–29.9)	29.6 (24.4–32)	0.874/0.874
PANNS W0	95 (94–98)	68 (62.8–74.2)	0.007/0.047
PANNS N subscale W0	28 (23–28)	20.5 (17–22.2)	0.020/0.070
PANNS P subscale W0	24 (23–26)	20 (15.8–22)	0.026/0.073
PANNS G subscale W0	46 (43–47)	32 (27.8–37)	0.010/0.047
SF36 W0	90 (83–97)	76.5 (72.5–83.8)	0.102/0.238
CGI-S W0	7 (6–7)	5 (5–6)	0.009/0.047
BMI (kg/m2)	−0.53 (−1.33–0.72)	0.35 (−0.23–0.90)	0.562/0.656
PANNS	−44 (−65–−31)	−37 (−39.5–−21.8)	0.342/0.749
PANNS N subscale	−10 (−17–−9)	−6 (−8.75–−4.5)	0.205/0.410
PANNS P subscale	−12 (−18–−6)	−11 (−15–−7.75)	0.874/0.874
PANNS G subscale	−22 (−26–−16)	−16 (−17.8–−8.75)	0.315/0.479
SF36	−5 (−18–−4)	−3 (−12.2–6)	0.245/0.429
CGI-I	4 (3–4)	3.5 (3–4)	0.452/0.575
**Variable (Males)**	**Cluster Type 2 (*n* = 5)**	**Cluster Type 3 (*n* = 4)**	***p*/*q*^a^**
BMI (kg/m^2^) W0	28.7 (27–29.9)	30.5 (27.5–32.2)	0.713/0.768
PANNS W0	95 (94–98)	67 (59.2–76.5)	0.037/0.198
PANNS N subscale W0	28 (23–28)	20 (18.8–22.2)	0.084/0.198
PANNS P subscale W0	24 (23–26)	17 (14–19)	0.027/0.198
PANNS G subscale W0	46 (43–47)	30.5 (24.8–37.5)	0.065/0.198
SF36 W0	90 (83–97)	83.5 (76.2–90)	0.391/0.547
CGI-S W0	7 (6–7)	5.5 (5–6)	0.050/0.198
BMI (kg/m^2^)	−0.53 (−1.33–0.72)	−0.92 (−1.97–−0.30)	0.713/0.768
PANNS	−44 (−65–−31)	−20.5 (−26.2–−17.8)	0.140/0.280
PANNS N subscale	−10 (−17–−9)	−6 (−7.5–−5.75)	0.389/0.547
PANNS P subscale	−12 (−18–−6)	−7.5 (−8.25–−6)	0.389/0.547
PANNS G subscale	−22 (−26–−16)	−8.5 (−11–−7)	0.085/0.198
SF36	−5 (−18–−4)	−11 (−13.5–−7)	1.0/1.0
CGI-I	4 (3–4)	4 (4–4)	0.661/0.768

^a^ Two-sided Wilcoxon rank-sum test, median with lower and upper quartiles in parentheses; BMI, PANNS, and SF36—changes from baseline (W0); CGI-I—an improvement from baseline; KEGG, Kyoto Encyclopedia of Genes and Genomes; BMI, body mass index; PANNS, positive and negative syndrome scale; SF36, 36- item short form survey; CGI, clinical global impression-improvement scale.

**Table 3:** Potential relationships between predicted metabolic changes and the severity of symptoms in schizophrenia (SZ) patients.

Pathways	Physiological Function	Potential Roles in SZ	References
Pathways found to be more active in patients with significantly less severe symptoms (according to PANNS and CGI-S)			
ko00430: Taurine and hypotaurine metabolism	Taurine: N-methyl-D-aspartate (NDMA) receptor inhibition and stem cell activation; a neurotransmitter and an inhibitory neuromodulator in the central nervous system (CNS); a potential immunomodulating compound, and an attenuator of apoptosis	Taurine supplementation was found to alleviate SZ symptoms significantly	[46,47,48]
Ko00250: Alanine (ALA), aspartate (ASP), and glutamate metabolism	ALA: An agonist that binds to the glycine site of NMDA receptors and improves the positive and cognitive symptoms of patients with SZ; ASP: Binding to the agonist site of NMDARs	NMDAR hypofunction in schizophrenia pathogenesis	[49,50]
Ko00790: Folate biosynthesis	Folate: Production of adenosylmethionine (SAM)	Schizophrenia patients may have lower folate levels (negative correlation with negative symptoms of SZ)	[51,52,53]
Ko00130: Ubiquinone and other terpenoids–quinone biosynthesis	Ubiquinone: ATP production, mitochondrial function, and reduction of proinflammatory mediators	Mitochondrial dysfunction as a part of SZ etiology	[54,55]
Ko00020: Citrate cycle (TCA cycle)	TCA: Normal energy metabolism of the brain	Abnormalities in energy metabolism were found to play a role in SZ pathophysiology	[56]
Ko00600: Sphingolipid (SL) metabolism	Formation of membrane “lipid rafts” of myelin sheaths, especially in neurons and oligodendrocytes (crucial for normal synaptic neurotransmission, axon-myelin stability, and communication/connectivity)	Inflammatory, synaptic, and white matter changes that result in disconnectivity in SZ may be related to SL	[57,58]
Pathways found to be more active in patients with significantly more severe symptoms (according to PANNS and CGI-S)			
Ko00030: Pentose phosphate pathway	Formation of NADPH for biosynthetic processes, cellular redox balance, and synthesis of ribose	Pentose phosphate pathway-related molecules in schizophrenia were found to be increased	[59]
Ko00061: Fatty acid biosynthesis	Component of membranes and myelination process mediator	Lipolysis and β-oxidation were found to be upregulated in SZ, as a result of insufficient brain energy supply	[60,61]
Ko00290: Valine, leucine, and isoleucine biosynthesis	Protein synthesis, production of energy, compartmentalization of glutamate synthesis of amine neurotransmitters, including serotonin, dopamine, and norepinephrine	Branched- chain amino acids when administered to patients with tardive dyskinesia—aberration of voluntary motor control in SZ patients treated with psychotropic drugs	[62,63]
Ko00072: Synthesis and degradation of ketone bodies	An alternative source of energy under fasting and starving; restrictive diets prolonged intense exercise	Ketones may change the ratio of GABA (glutamate in favor of GABA) to compensate GABA levels in the CNS in SZ patients	[64]

PANNS—The Positive and Negative Syndrome Scale, CGI-S—The Clinical Global Impressions Scale.

## Supplementary Materials

The following are available online at https://www.mdpi.com/2077-0383/8/10/1605/s1.
