# Supplementary material for: Analysis of Gut Microbiota and Their Metabolic Potential in Patients with Schizophrenia Treated with Olanzapine: Results from a Six-Week Observational Prospective Cohort Study
Source: J Clin Med. 2019 Oct 3;8(10):1605. doi: 10.3390/jcm8101605 (PMC6832832; doi:10.3390/jcm8101605)
Supplement: Supplementary file 1 [file jcm-08-01605-s001.pdf]

Supplementary data

Supplementary Table S1. Average daily macronutrient intake during the hospital stay.

Variables	Recommended Daily Intake [a]	Result	
		Mean	Range
Energy, kcal		2995	2865–3165
Protein, g		106	89–122
Fat, g		102	91–116
Carbohydrates, g		420	387–442
Fiber, g	>25	31	28–33
% Energy from protein	10–20	14	12–15
% Energy from fat	20–35	31	29–33
% Energy from carbohydrates	45–65	56	54–56

[a] For healthy Polish people [1].

Supplementary Table S2. Correlations of PC1 changes with demographic, clinical, and environmental factors.

Variables	rho	*p*/*q* [b]
Age (years)	−0.26	0.275/0.756
BMI (kg/m^2)	−0.18	0.437/0.867
Olanzapine average dose per day (mg)	0.11	0.649/0.976
Olanzapine maximum dose (mg)	0.38	0.098/0.359
Disease duration (months)	0.05	0.820/0.976
Duration of untreated psychosis (months)	−0.01	0.954/0.976
Smoking (number of cigarettes per day) [a]	−0.007	0.976/0.976
Coffee (number of cups)	−0.40	0.083/0.359
Tea (number of cups)	0.02	0.938/0.976

[a] Ordinal variables (per day): 1, non-smokers; 2, up to 10 cigarettes; 3, up to 20 cigarettes; 4, up to 40 cigarettes; rho, Spearman correlation coefficient; [b] *p*, two-sided Wilcoxon rank-sum test; *q*, FDR adjusted *p*-value.

Supplementary Table S3. Clinical scales at baseline (W0) and end-point (W6).

Scales	W0	W6	*p*/*q* [a]
PANNS total score	74 (63.8–93.2)	45.5 (29.8–54.8)	0.0001/0.0002
PANNS N subscale	21.5 (18.8–24.2)	13 (9–15.2)	0.0002/0.0002
PANNS P subscale	20.5 (17.5–23.3)	9.5 (4.75–12.2)	0.0001/0.0002
PANNS G subscale	34 (31.2–43.2)	23 (14.5–26.5)	0.00009/0.0002
SF36	82 (73.8–89.2)	77 (71.2–80.2)	0.027/0.027
CGI-S	6 (5–6)	4 (3–4)	0.00006/0.0002

PANNS: Positive and Negative Syndrome Scale; SF36: Short Form (36) Health Survey; CGI-S: Clinical Global Impression—Severity; [a] *p*: two-sided Wilcoxon signed-rank test; *q*: FDR adjusted *p*-value.

Supplementary Table S4. Correlation of changes in clinical scales with demographic and clinical characteristics.

	Age	BMI	Olanzapine Maximum Dose	Olanzapine Average Dose	Disease Duration	Duration of Untreated Disease
PANNS total score	−0.17 (0.476/0.559)	0.24 (0.305/0.492)	0.45 (0.047/0.120)	0.26 (0.269/0.497)	0.31 (0.191/0.382)	0.66 (0.002/0.003)
PANNS negative	0.20 (0.408/0.559)	0.16 (0.492/0.492	0.15 (0.537/0.712)	0.07 (0.784/0.941)	0.05 (0.835/0.835)	0.68 (0.001/0.003)
PANNS positive	−0.33 (0.156/0.559)	0.31 (0.187/0.492	0.43 (0.060/0.120)	0.24 (0.311/0.497)	0.50 (0.024/0.144)	0.48 (0.030/0.037)
PANNS general	−0.14 (0.559/0.559)	0.19 (0.427/0.492	0.43 (0.059/0.120)	0.29 (0.214/0.497)	0.26 (0.273/0.394)	0.65 (0.002/0.003)
SF36	−0.17 (0.474/0.559)	−0.46 (0.044/0.264	−0.08 (0.750/0.750)	0.01 (0.957/0.957)	−0.44 (0.056/0.168)	0.11 (0.651/0.651)
CGI-S	−0.18 (0.442/0.559)	0.18 (0.451/0.492	0.13 (0.593/0.712)	0.23 (0.331/0.497)	0.23 (0.328/0.394)	0.68 (0.0009/0.003)

[a] rho, Spearman correlation coefficient; p, two-sided Wilcoxon rank-sum test; q, FDR adjusted p-value.

Supplementary Table S5. Association of OTU s′ genus-level types with responses to treatment (PANNS, SF36, and CGI).

**Scale (Females + Males)**	**Cluster Type1 (*n* = 9)**	**Cluster Type2 (*n* = 10)**	***p*/*q*** [a]
BMI W0	29.1 (26.0–32.0)	26.1 (24.3–30.7)	0.488/0.683
PANNS W0	70 (64–84)	83.5 (64.2–94.5)	0.462/0.683
PANNS N subscale W0	21 (19–22)	23 (15.8–25.8)	0.486/0.683
PANNS P subscale W0	18 (15–22)	22 (20.2–24)	0.164/0.683
PANNS G subscale W0	33 (29–43)	38 (32.3–45.8)	0.251/0.683
SF36 W0	82 (73–91)	80 (75–82.8)	0.838/0.902
CGI-S W0	5 (5;6)	6 (5.25–6)	0.483/0.683
BMI (kg/m^2)	0 (-1.33–0.64)	0.43 (−0.23–1.38)	0.307/0.683
PANNS	−38 (-51–−23)	−33.5 (−40.5–−19.2)	0.567/0.722
PANNS N subscale	−8 (-11–−6)	−6.5 (−9.75–−1.5)	0.346/0.683
PANNS P subscale	−11 (-15–−9)	−7 (−15.8–−6.3)	0.486/0.683
PANNS G subscale	−15 (-26–−9)	−16.5 (−19.2–−8.3)	0.743/0.867
SF36	−8 (−13–6)	−4.5 (−12.2–−1)	0.902/0.902
CGI-I	4 (3–4)	4 (3–4)	0.450/0.683
Scale (Females)	Cluster Type1 (*n* = 5)	Cluster Type2 (*n* = 4)	*p*/*q* [a]
BMI (kg/m^2) W0	26.0 (24.5–30.1)	28.0 (24.7–31.4)	0.903/1.0
PANNS W0	70 (66–84)	61 (59.2–64.2)	0.111/0.758
PANNS N subscale W0	21 (20–22)	13 (10–16.2)	0.325/0.758
PANNS P subscale W0	20 (15–22)	20.5 (18–21.8)	1.0/1.0
PANNS G subscale W0	33 (32–43)	30 (27.8–32.3)	0.174/0.758
SF36 W0	75 (73–82)	76 (73–79)	1.0/1.0
CGI-S W0	5 (5–6)	5 (5–5.25)	0.661/1.0
BMI (kg/m^2)	0.64 (0.57–0.70)	1.24 (0.76–1.78)	0.391/0.782
PANNS	−39 (−51–−38)	−25 (−39.5–−9)	0.325/0.758
PANNS N subscale	−8 (−11–−8)	−0.5 (−2–0.25)	0.037/0.518
PANNS P subscale	−15 (−15–−11)	−12 (−17.8–−5.25)	1.0/1.0
PANNS G subscale	−17 (−28–−15)	−10.5 (−17.8–−4)	0.323/0.758
SF36	6 (−8–6)	0.5 (−6.75–5.25)	0.533/0.933
CGI-I	3 (3–4)	3 (3–3.5)	0.893/1.0
Scale (Males)	Cluster Type1 (*n* = 4)	Cluster Type2 (*n* = 6)	*p*/*q* [a]
BMI (kg/m^2) W0	30.5 (29.0–32.3)	26.1 (23.3–29.1)	0.241/0.520
PANNS W0	67 (59.2–76.8)	94 (90.8–97.2)	0.070/0.327
PANNS N subscale W0	20 (18.8–22.8)	25.5 (23.5–27.5)	0.163/0.465
PANNS P subscale W0	17 (14–19.2)	23 (22–25.5)	0.069/0.327
PANNS G subscale W0	30.5 (24.8–37)	45.5 (43.5–46.8)	0.054/0.327
SF36 W0	91 (84.5–94.8)	82.5 (79–88.2)	0.594/0.644
CGI-S W0	5.5 (5–6.25)	6 (6–6.75)	0.298/0.520
BMI (kg/m^2)	−1.42 (−1.97–−1.08)	−0.22 (−0.46–0.49)	0.166/0.465
PANNS	−20.5 (−33.5–−17.8)	−33.5 (−42–−25)	0.334/0.520
PANNS N subscale	−6 (−9.8–−5.8)	−9.5 (−11.5–−8.3)	0.593/0.644
PANNS P subscale	−8.5 (−11.2–−6.8)	−7 (−10.8–−6.3)	0.915/0.915
PANNS G subscale	−8.5 (−13.2–−7)	−16.5 (−20.8–−10.8)	0.334/0.520
SF36	−15 (−18.8–−8.5)	−7.5 (−12.2–−4.3)	0.521/0.644
CGI-I	4 (3.8–4)	4 (4–4)	0.598/0.644

[a] two-sided Wilcoxon rank-sum test; q, FDR adjusted p-value. Median with lower and upper quartiles in parentheses; PANNS (total score), PANNS N subscale, PANNS P subscale, PANNS G subscale, and SF36: changes from baseline (W0); CGI-I: an improvement from baseline.

Supplementary Table S6. Associations of KEGG orthologs with BMI changes and clinical improvements (PANNS, SF36, and CGI).

**Variable (Females + Males)**	**Cluster Type1 (*n* = 7)**	**Cluster Type2 (*n* = 13)**	***p*/*q*** [a]
BMI (kg/m^2) W0	25.3 (24.7–31.5)	29.1 (26.0–31.0)	0.874/1.0
PANNS W0	71 (64–77.5)	84 (64–95)	0.322/0.632
PANNS N subscale W0	18 (13–24.5)	22 (21–24)	0.361/0.632
PANNS P subscale W0	19 (18–21.5)	22 (16–24)	0.301/0.632
PANNS G subscale W0	33 (32.5–35.5)	43 (29–44)	0.662/0.927
SF36 W0	82 (78–85)	82 (73–90)	0.937/1.0
CGI-S W0	6 (5.5–6)	6 (5–7)	0.898/1.0
BMI (kg/m^2)	−0.19 (−0.29–0.55)	0.57 (−1.33–0.72)	1.0/1.0
PANNS	−24 (−31.5–−23)	−39 (−51–−18)	0.165/0.632
PANNS N subscale	−5 (−6.5–−4)	−8 (−11–-6)	0.164/0.632
PANNS P subscale	−7 (−10–−7)	−12 (−15–-7)	0.264/0.632
PANNS G subscale	−12 (−15–−8.5)	−17 (−26–-9)	0.176/0.632
SF36	−13 (−14.5–−7)	−4 (−12–5)	0.283/0.632
CGI-I	4 (3.5–4)	4 (3–4)	0.409/0.636
Variable (Females)	Cluster Type1 (*n* = 3)	Cluster Type2 (*n* = 6)	*p*/*q* [a]
BMI (kg/m^2) W0	24.9 (24.7–28.6)	28.1 (24.6–30.8)	1.0/1.0
PANNS W0	62 (59.5–64)	70.5 (65.5–80.8)	0.156/0.680
PANNS N subscale W0	12 (12–13)	21.5 (20.2–22.8)	0.154/0.680
PANNS P subscale W0	21 (16.5–21.5)	20 (16.2–23)	0.896/1.0
PANNS G subscale W0	32 (29.5–32.5)	32.5 (29.8–40.5)	0.515/1.0
SF36 W0	78 (73–80)	74.5 (73.2–80.2)	1.0/1.0
CGI-S W0	5 (5–5.5)	5 (5–5.75)	1.0/1.0
BMI (kg/m^2)	0.97 (0.55–1.58)	0.67 (0.59–1.31)	0.897/1.0
PANNS	−27 (−33–−15)	−40 (−48.5–−38.2)	0.195/0.680
PANNS N subscale	−3 (−4–−1)	−8 (−10.2–−2.75)	0.243/0.680
PANNS P subscale	−11 (−14–−5.5)	−15 (−15–−12)	0.598/1.0
PANNS G subscale	−13 (−15–−8.5)	−18.5 (−26–−15.5)	0.241/0.680
SF36	−4 (−9.5–4.5)	5.5 (−4.75–6)	0.896/1.0
CGI-I	3 (3;4)	3 (3–3.75)	0.670/1.0
Variable (Males)	Cluster Type1 (*n* = 4)	Cluster Type2 (*n* = 7)	*p*/*q* [a]
BMI (kg/m^2) W0	28.0 (24.6–31.3)	29.1 (27.8–30.9)	0.925/0.925
PANNS W0	77.5 (75.5–81.8)	94 (76.5–96.5)	0.395/0.925
PANNS N subscale W0	24.5 (22.5–25.2)	23 (21–28)	0.776/0.925
PANNS P subscale W0	18.5 (18–19.8)	23 (19–25)	0.343/0.925
PANNS G subscale W0	35.5 (34.5–38.2)	43 (34.5–46.5)	0.507/0.925
SF36 W0	85 (81–89.2)	89 (77.5–93.5)	0.777/0.925
CGI-S W0	6 (6–6)	6 (5.5–7)	0.754/0.925
BMI (kg/m^2)	−0.29 (−0.60–−0.23)	−1.33 (−2.42–0.10)	0.508/0.925
PANNS	−23.5 (−27–−23)	−31 (−54.5–−18)	0.9240.925
PANNS N subscale	−6.5 (−9–−5)	−9 (−13.5–−6)	0.506/0.925
PANNS P subscale	−7 (−7.5–−7)	−8 (−15–−6)	0.924/0.925
PANNS G subscale	−10.5 (−13.2–−8.75)	−16 (−24–−8.5)	0.635/0.925
SF36	−13.5 (−15–−12.2)	−5 (−15–−2)	0.344/0.925
CGI-I	4 (4–4)	4 (3.5–4)	0.717/0.925

[a] two-sided Wilcoxon rank-sum test, median with lower and upper quartiles in parentheses; BMI, PANNS, and SF36: changes from baseline (W0); CGI-I, an improvement from baseline.

Supplementary Table S7. Association of KEGG modules with BMI changes and clinical improvements (PANNS, SF36, and CG1I).

**Variables (Females + Males)**	**Cluster Type 2 (*n* = 5)**	**Cluster Type 3 (*n* = 12)**	***p*/*q*** [a]
BMI (kg/m^2) W0	27 (26–31)	29.5 (24.1–32)	1.0/1.0
PANNS W0	71 (70–90)	77.5 (63.8–94.8)	0.958/1.0
PANNS N subscale W0	22 (21–23)	21 (18.8–26.5)	0.833/1.0
PANNS P subscale W0	22 (20–24)	21.5 (17.5–23.2)	0.791/1.0
PANNS G subscale W0	39 (28.5–43.2)	33 (32–46)	0.832/1.0
SF36 W0	73 (72–74)	82.5 (77.2–91.5)	0.672/1.0
CGI-S W0	5 (5–6)	6 (5–6.25)	0.126/0.882
BMI (kg/m^2)	0 (−0.53–1.51)	0.35 (−0.58–0.71)	1.0/1.0
PANNS	−41 (−44–−31)	−37 (−42–−21.8)	0.635/1.0
PANNS N subscale	−9 (−10–−1)	−7 (−11.2–−5)	0.874/1.0
PANNS P subscale	−12 (−15–−7)	−11 (−15.5–−7.75)	1.0/1.0
PANNS G subscale	−20 (−22–−16)	−16 (−19.2–−9)	0.525/1.0
SF36	5 (0–6)	−11 (−15.8–−2.5)	0.064/0.882
CGI-I	3 (3–4)	4 (3–4)	0.325/1.0
Variables (Females)	Cluster Type 2 (*n* = 3)	Cluster Type 3 (*n* = 5)	*p*/*q* [a]
BMI (kg/m^2) W0	26 (25.1–28.5)	30.1 (24.5–32.4)	0.766/0.975
PANNS W0	70 (65–70.5)	66 (64–84)	0.766/0.975
PANNS N subscale W0	22 (13–22.5)	20 (14–21)	1.0/1.0
PANNS P subscale W0	20 (17.5-22)	21 (20–22)	0.763/0.975
PANNS G subscale W0	32 (30–32.5)	32 (29–43)	0.881/1.0
SF36 W0	73 (71.5–73.5)	82 (75–82)	0.230/0.975
CGI-S W0	5 (5–5)	5 (5–6)	0.329/0.975
BMI (kg/m^2)	1.51 (0.76–2.04)	0.64 (0.57–0.70)	0.766/0.975
PANNS	−41 (−55–−26)	−39 (−39–−38)	0.764/0.975
PANNS N subscale	−1 (−11.5–−0.5)	−8 (−8–−5)	0.549/0.975
PANNS P subscale	−15 (−17.5–−11)	−15 (015–−11)	1.0/1.0
PANNS G subscale	−20 (−26–−12)	−17 (−17–−15)	0.764/0.975
SF36	6 (5.5–6)	−8 (−13–6)	0.541/0.975
CGI-I	3 (2.5–3)	3 (3–4)	0.168/0.975

[a] two-sided Wilcoxon rank-sum test, median with lower and upper quartiles in parentheses; BMI, PANNS, and SF36: changes from baseline (W0); CGI-I, an improvement from baseline.

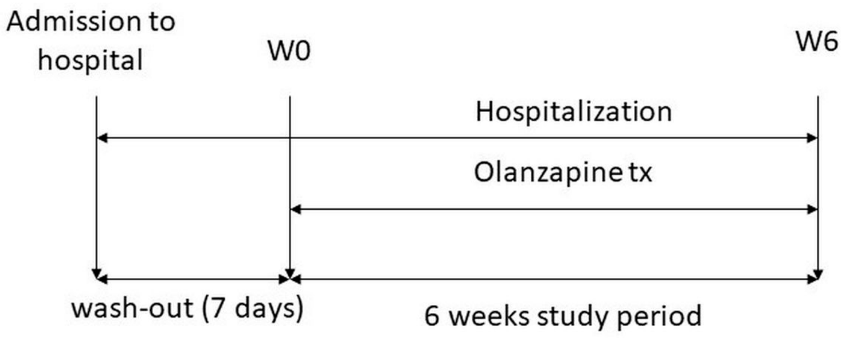

Supplementary Figure S1. Study schema. Tx, treatment; W0, after the washout period; W6, after 6 weeks of treatment.

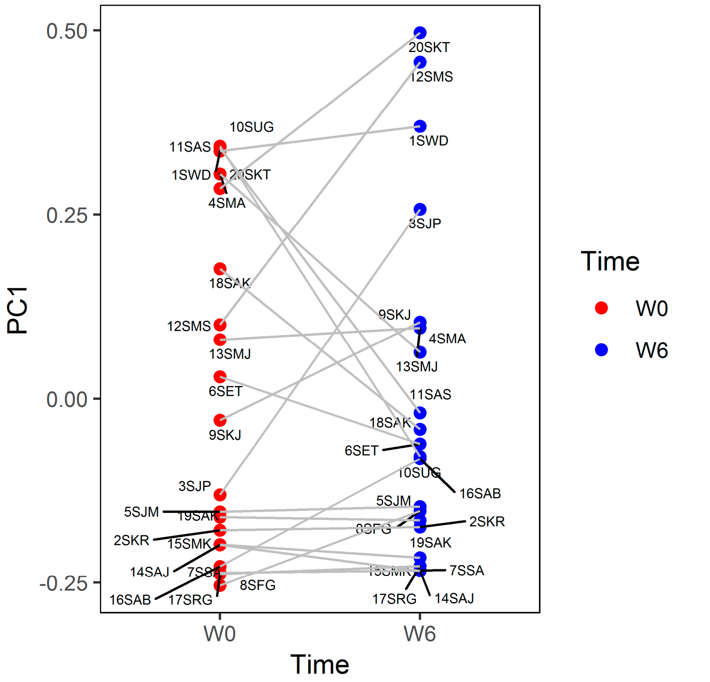

Supplementary Figure S2. Changes in the PC1 in patients between W0 and W6.

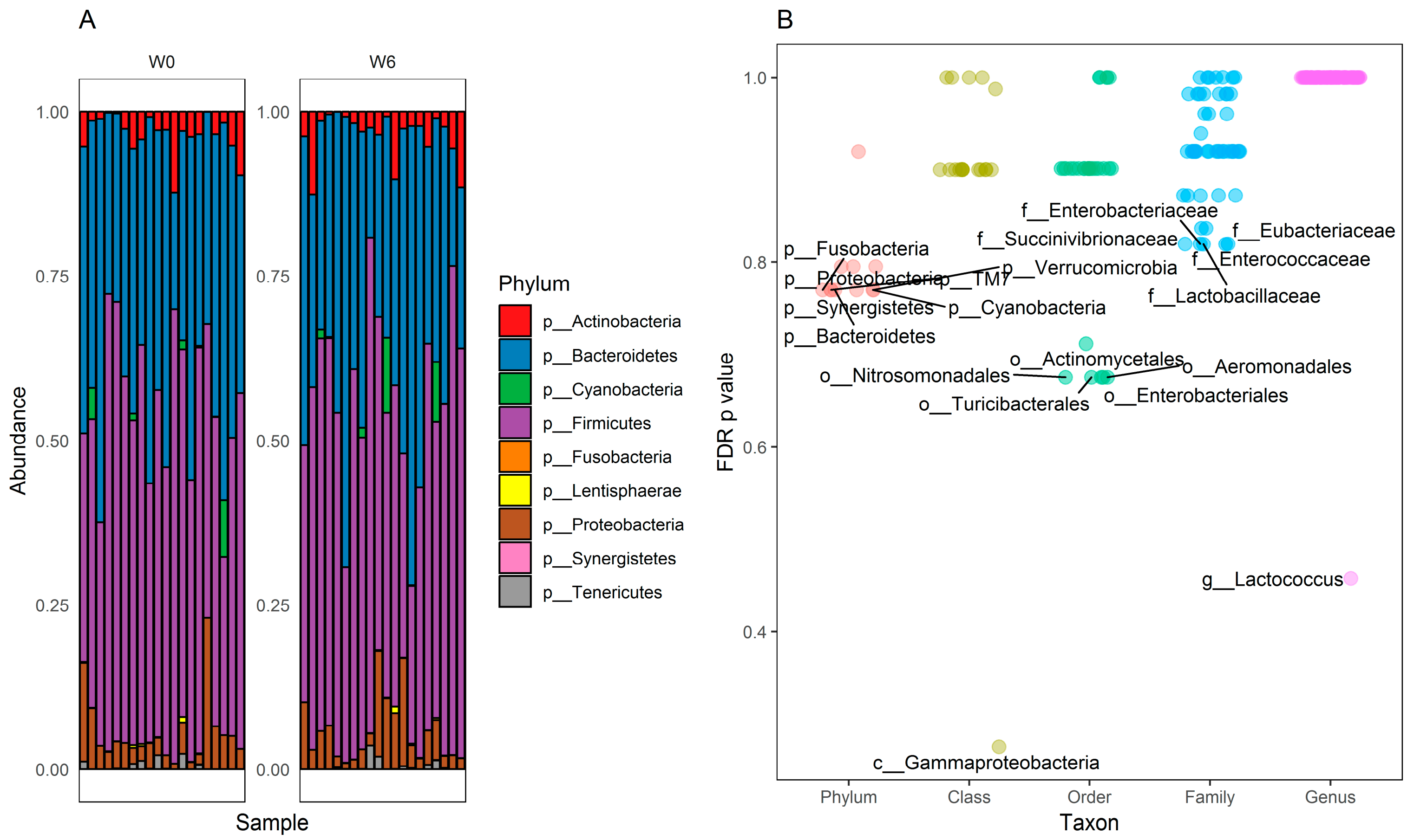

Supplementary Figure S3. Differential abundance testing between first (W0) and end-point (W6) samples (gut microbiome compositions). A—stacked barplot of phylogenic compositions of bacterial taxa at the phylum level (9 most abundant Phyla are shown) by the time of collection (W0 vs. W6). B—FDR adjusted p values from differential abundance testing (W0 vs. W6) at the phylum, class, order, family, and genus levels using the Wilcoxon signed-rank test. Only labels for the minimum FDR adjusted p values for each taxonomic level are shown.

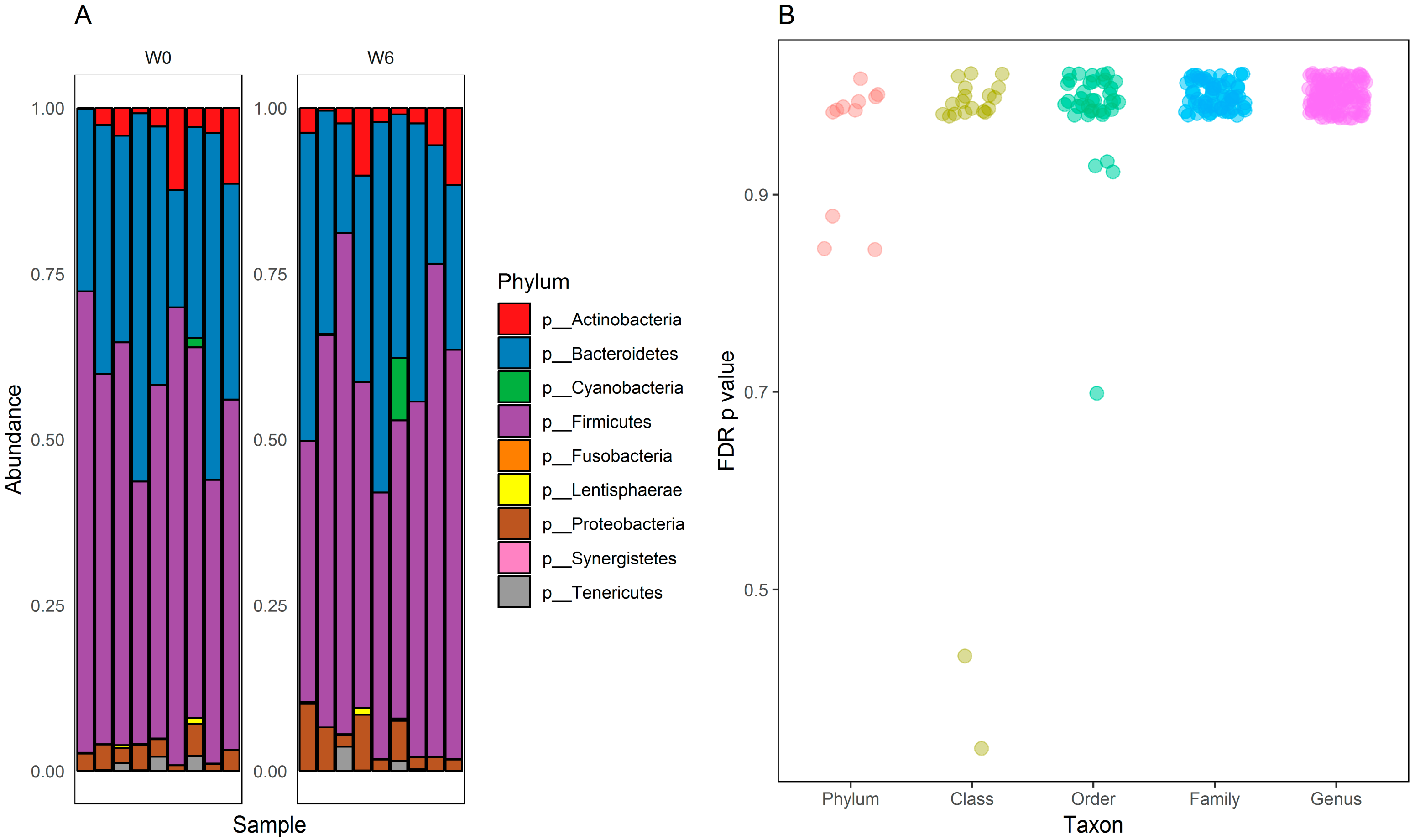

Supplementary Figure S4. Differential abundance testing between first (W0) and end-point (W6) samples (gut microbiome compositions) in females. A—stacked barplot of phylogenic compositions of bacterial taxa at the phylum level (9 most abundant Phyla are shown) by the time of collection (W0 vs. W6). B—FDR adjusted p values from differential abundance testing (W0 vs. W6) at the phylum, class, order, family, and genus levels using the Wilcoxon signed-rank test. Only labels for the minimum FDR adjusted p values for each taxonomic level are shown.

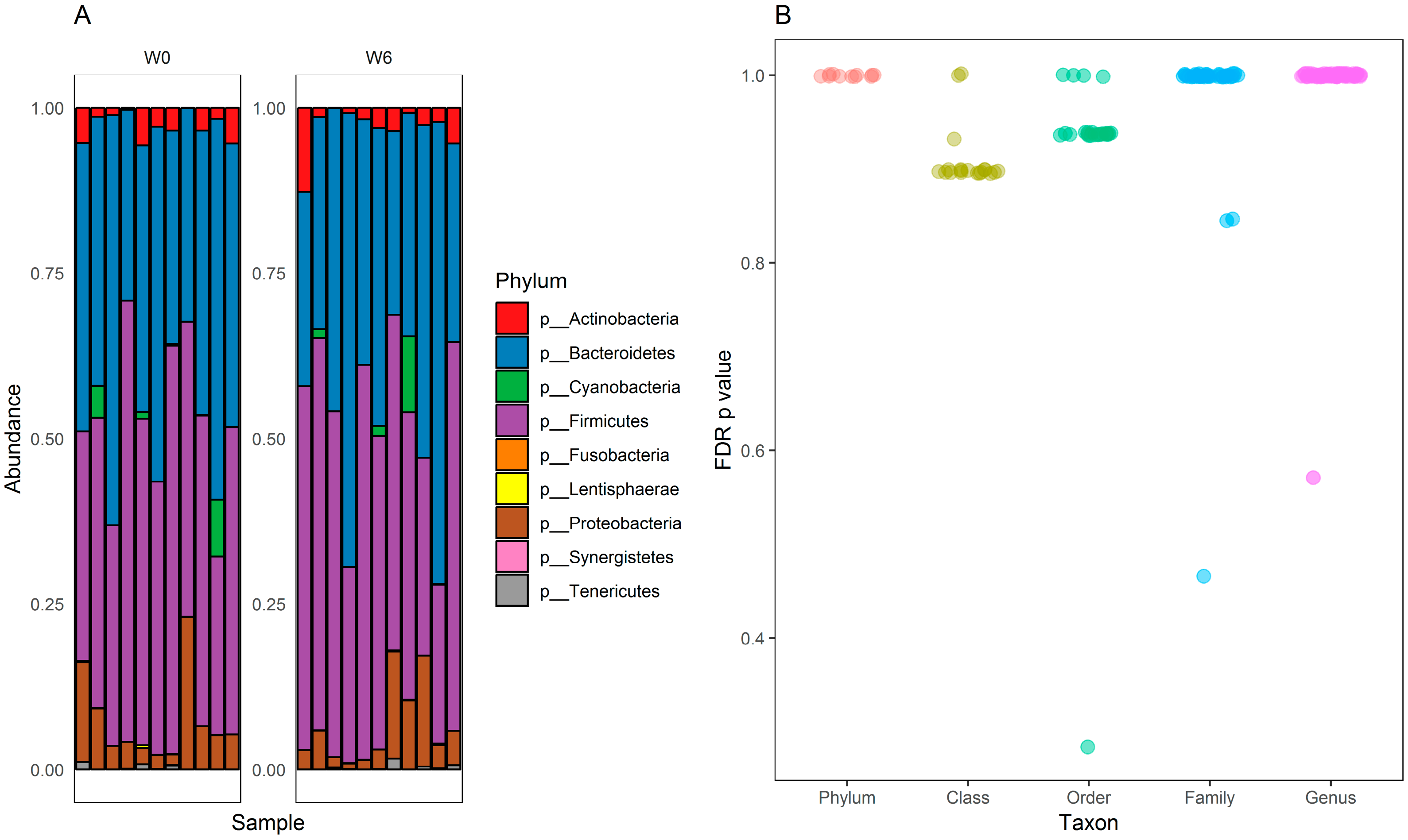

Supplementary Figure S5. Differential abundance testing between first (W0) and endpoint (W6) samples (gut microbiome compositions) in males. A—stacked bar plots of the phylogenic composition of bacterial taxa at the phylum level (9 most abundant Phyla are shown) by the time of collection (W0 vs. W6). B—FDR adjusted p values from differential abundance testing (W0 vs. W6) at the phylum, class, order, family, and genus levels using the Wilcoxon signed-rank test. Only labels for the minimum FDR adjusted p values for each taxonomic level are shown.

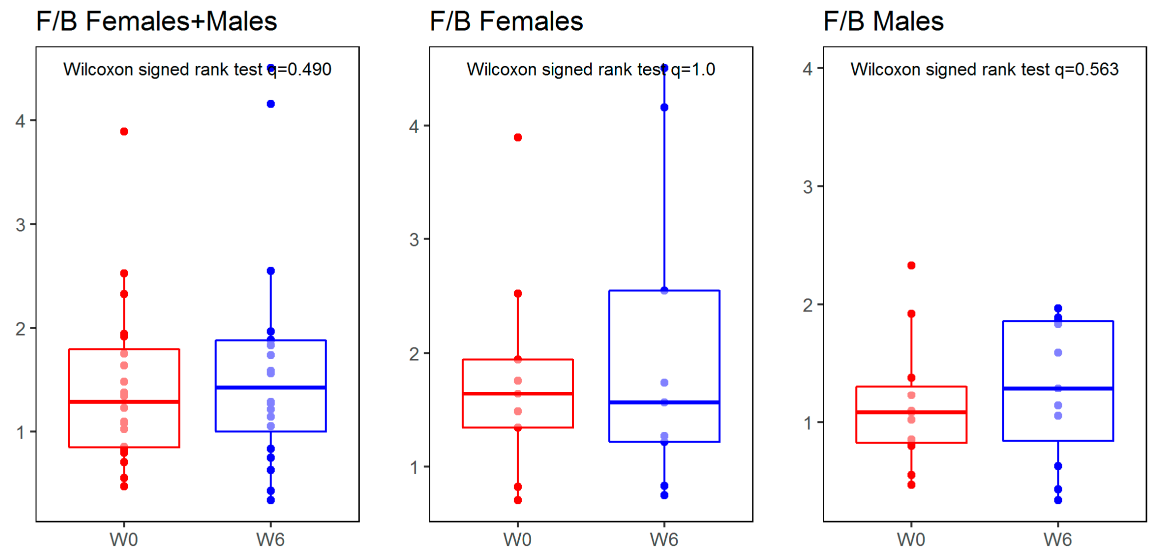

Supplementary Figure S6. The boxplot shows the F/B (Firmicutes to Bacteroidetes ratio) at baseline (W0) and after 6 weeks (W6). FDR adjusted p values (q) are shown (two-sided). Center line: median, lower, and upper hinges correspond to the first [Q1] and third [Q3] quartiles; Whiskers: the upper whisker is located at the smaller of the maximum Bray–Curtis measures and Q3 + 1.5 * IQR (Q3–Q1); the lower whisker is located at the larger of the minimum Bray–Curtis measures and Q1—1.5 * IQR). W0 and W6 represent different time points.

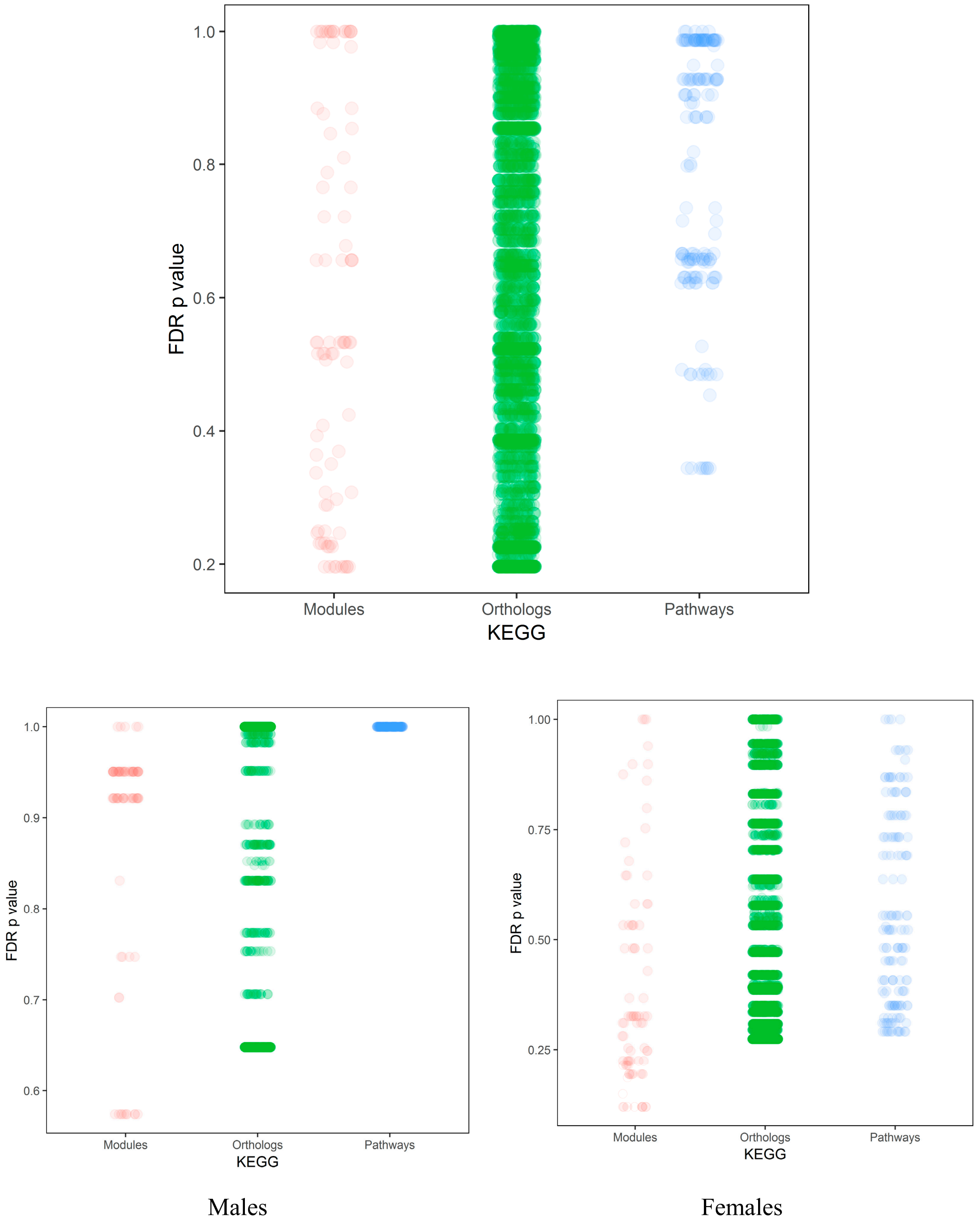

Supplementary Figure S7. Differential abundance testing between first (W0) and end-point (W6) samples (KEGG Orthologs, Modules, and Pathways). FDR adjusted p values from differential abundance testing (W0 vs. W6) of the KEGG Orthologs, Modules, and Pathways using the Wilcoxon signed-rank test. KEGG Orthologs, Modules, and Pathways abundances were calculated from 16S rRNA sequencing data using PICRUSt and HUManN.

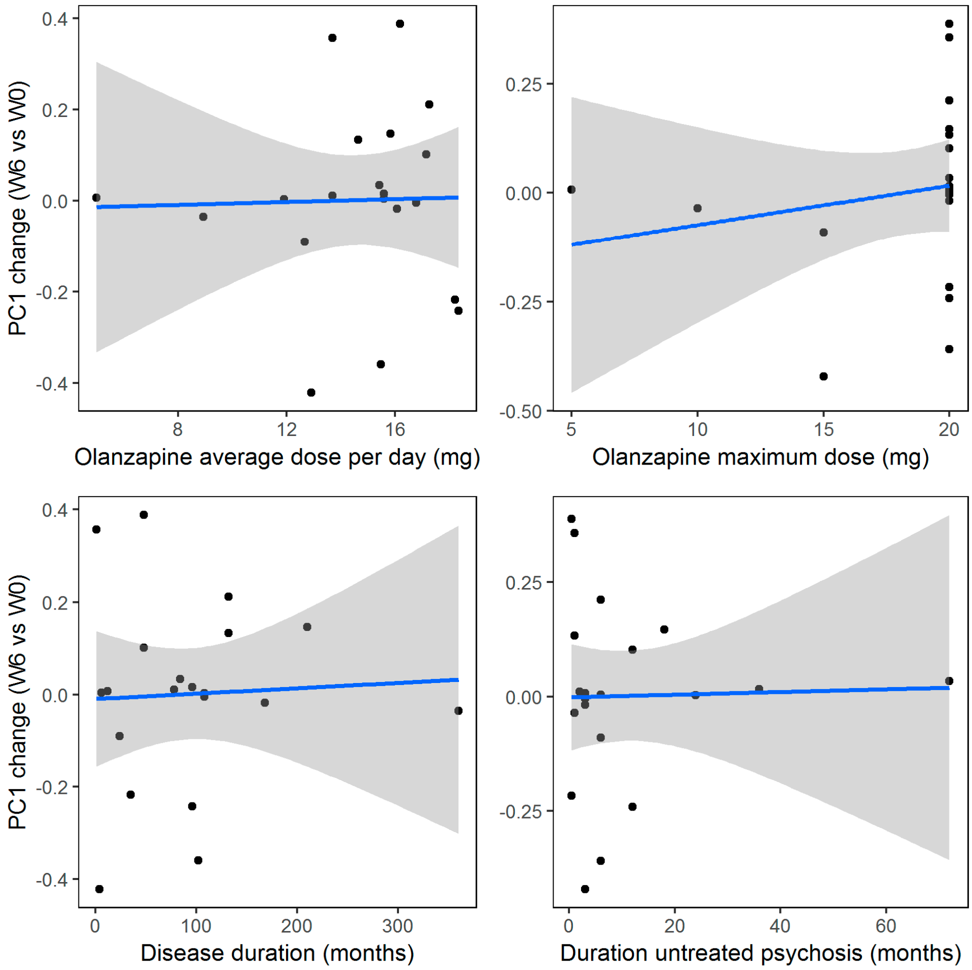

Supplementary Figure S8. Correlations of the Olanzapine dosage or disease duration with the PC1 change (W6 vs. W0). The regression lines (colored blue) were fitted using the linear model. Grey shading areas represent confidence bounds. PC1, principal coordinate 1.

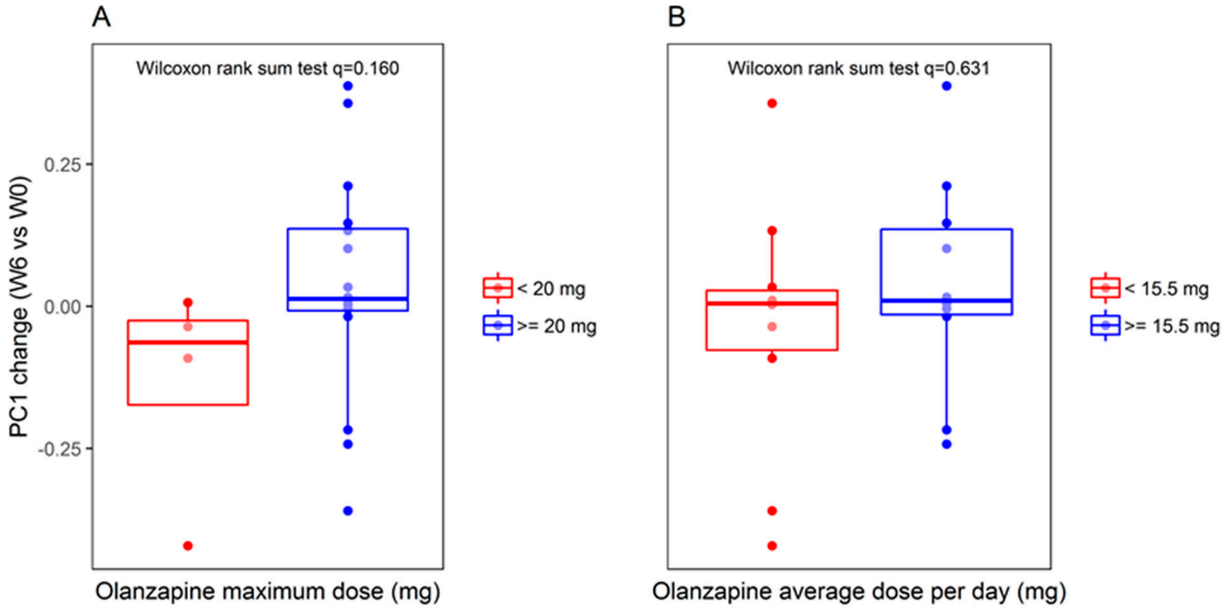

Supplementary Figure S9. Changes in the gut microbial community compositions (as measured by a shift in the PC1). (A) Olanzapine maximum dose < 20 (-0.06 [-0.17–-0.02]) vs ≥ 20 mg (0.01 [-0.01–0.14]). (B) Olanzapine average dose per day < 15.5 (0.01 [-0.08–0.03]) vs ≥ 15.5 mg (0.01 [-0.01–0.14]). FDR adjusted p values (q) are shown (two-sided). Center line: median, lower, and upper hinges correspond to the first [Q1] and third [Q3] quartiles. Whiskers: the upper whisker is located at the smaller of the maximum Bray–Curtis measures and Q3 + 1.5 * IQR (Q3–Q1).

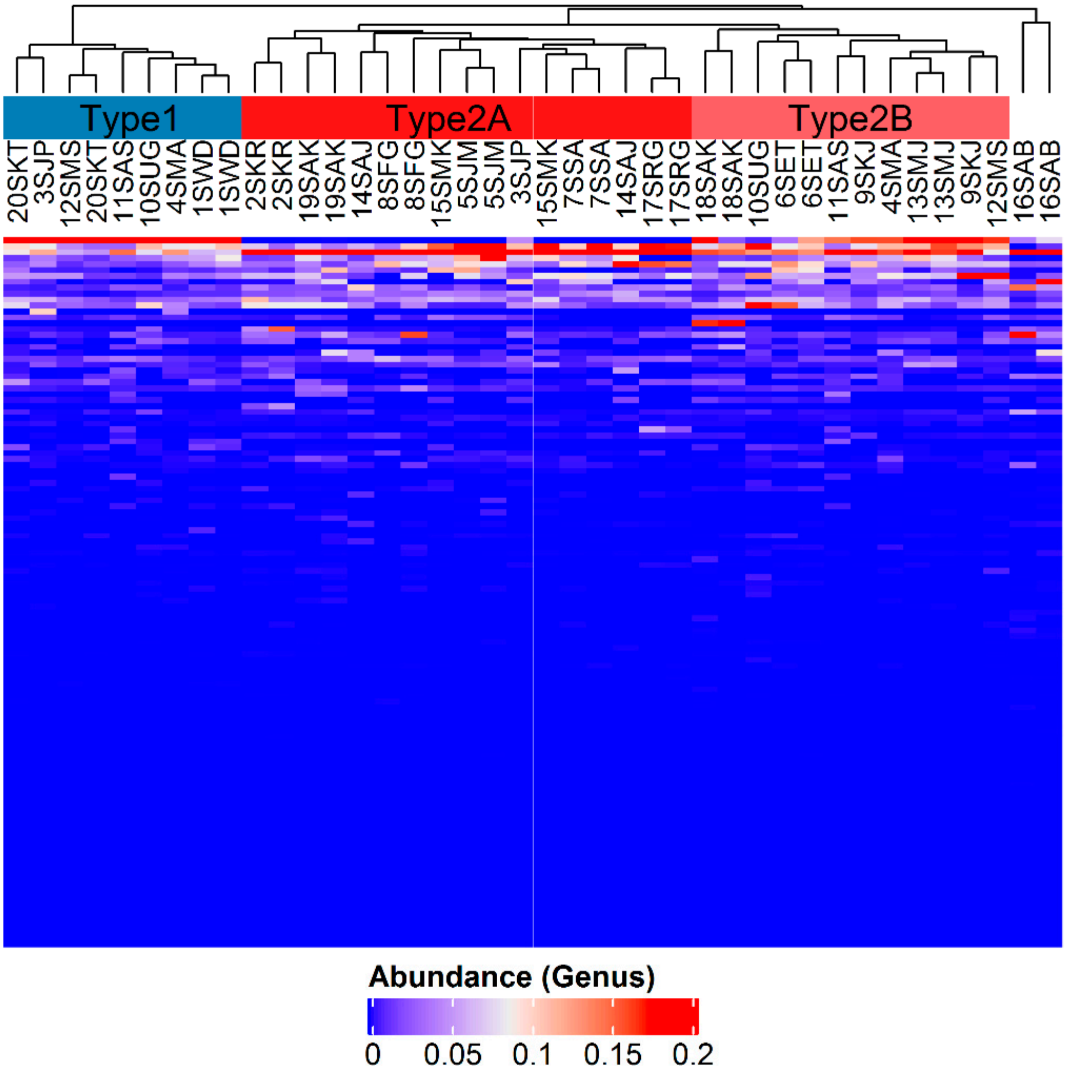

Supplementary Figure S10. Genus level resolution analysis of gut microbiota in patients diagnosed with paranoid schizophrenia treated with olanzapine during six weeks of hospitalization. Cluster Type 2 (FIgure 3A) was divided into two clusters (Type 2A nad Type 2B).

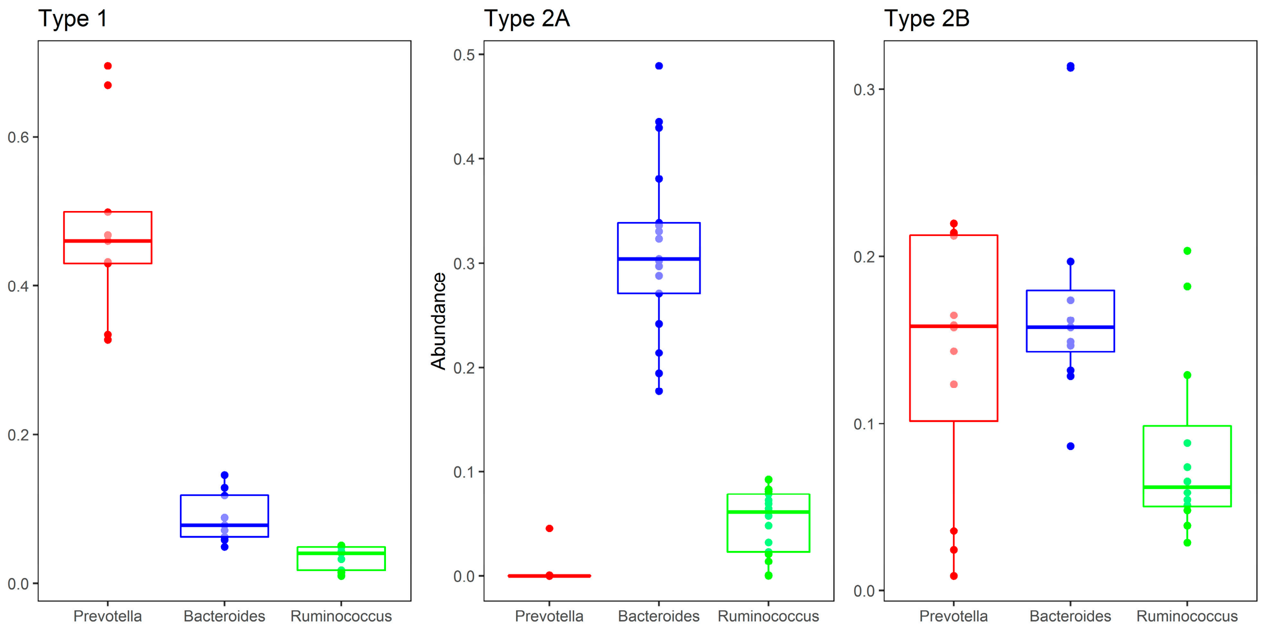

Supplementary Figure S11. The relative abundances of main contributors of each enterotype (*Bacteroides, Prevotella, Ruminococcus*) in the three clusters (Type 1, Type 2A, Type 2B). Main contributors of enterotypes according to Arumugam et al.[2].

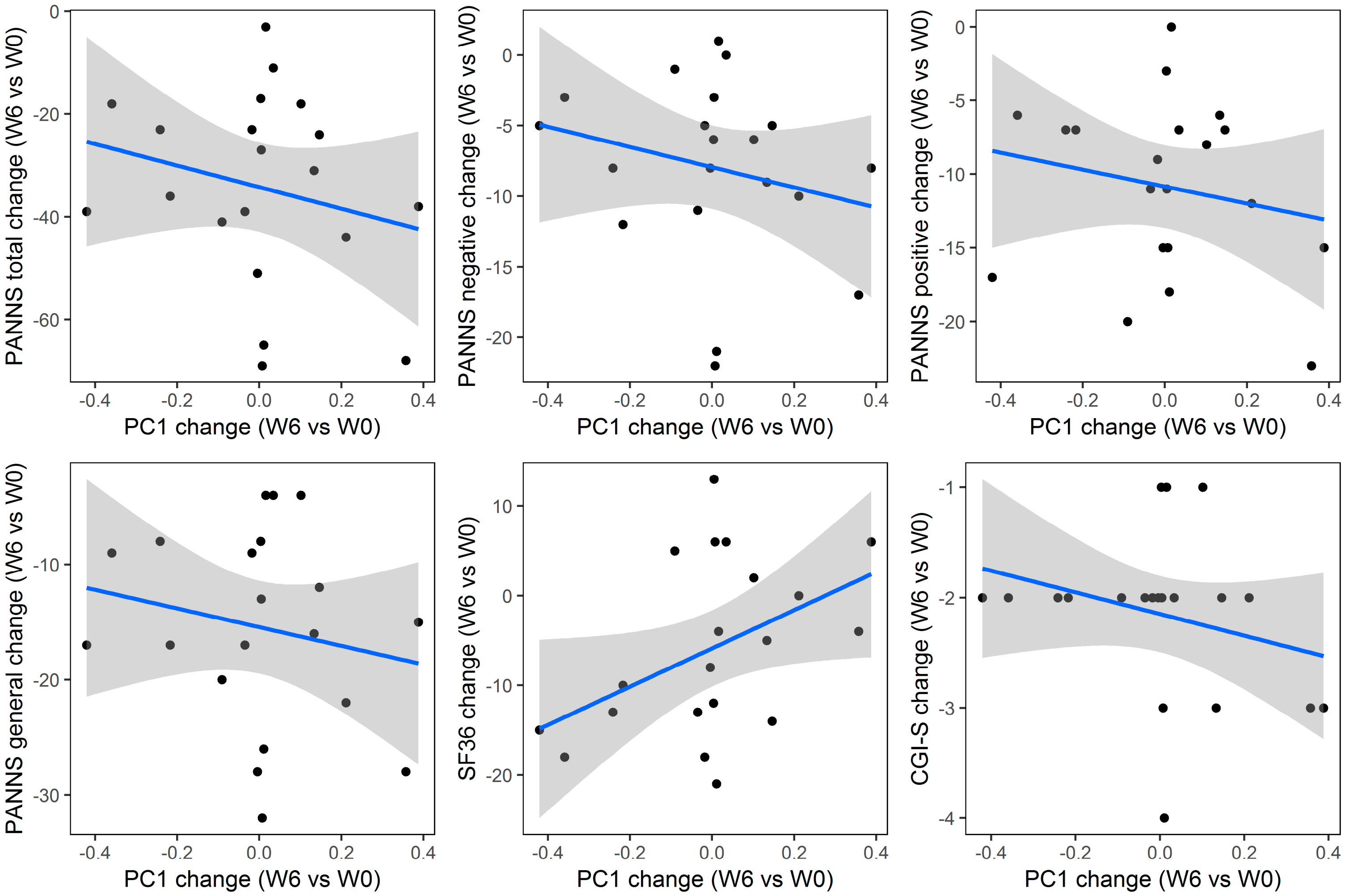

Supplementary Figure S12. Correlations of the change in the PC1 (W6 vs. W0) with the changes in the PANNS total score and negative, positive, or general subscales-SF36-or CGI-S. The regression lines (colored blue) were fitted using the linear model. Grey shading areas represent confidence bounds. PC1, principal coordinate 1.

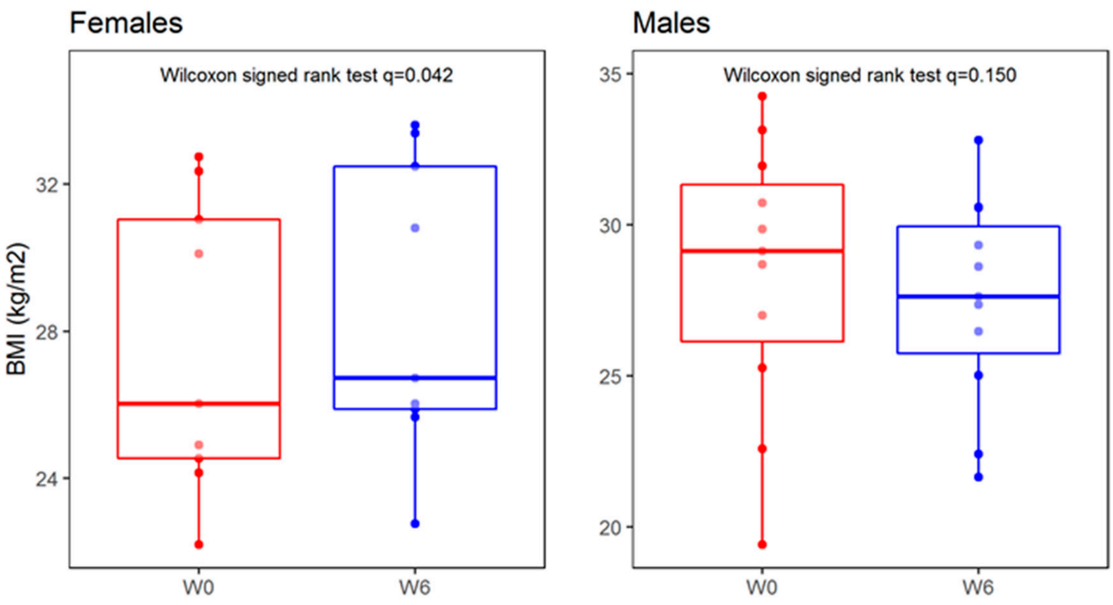

Supplementary Figure S13. BMI at baseline (W0) and after 6 weeks of treatment (W6). Females: 26.0 (24.5–31.0) (W0) vs 26.7 (25.9–32.5) (W6); Males: 29.1 (26.1–31.3) (W0) vs 27.6 (25.7–29.9) (W6). FDR adjusted p values (q) are shown (two-sided). Center line: median, lower, and upper hinges correspond to the first [Q1] and third [Q3] quartiles; whiskers: the upper whisker is located at the smaller of the maximum Bray–Curtis measures and Q3 + 1.5 * IQR (Q3–Q1).

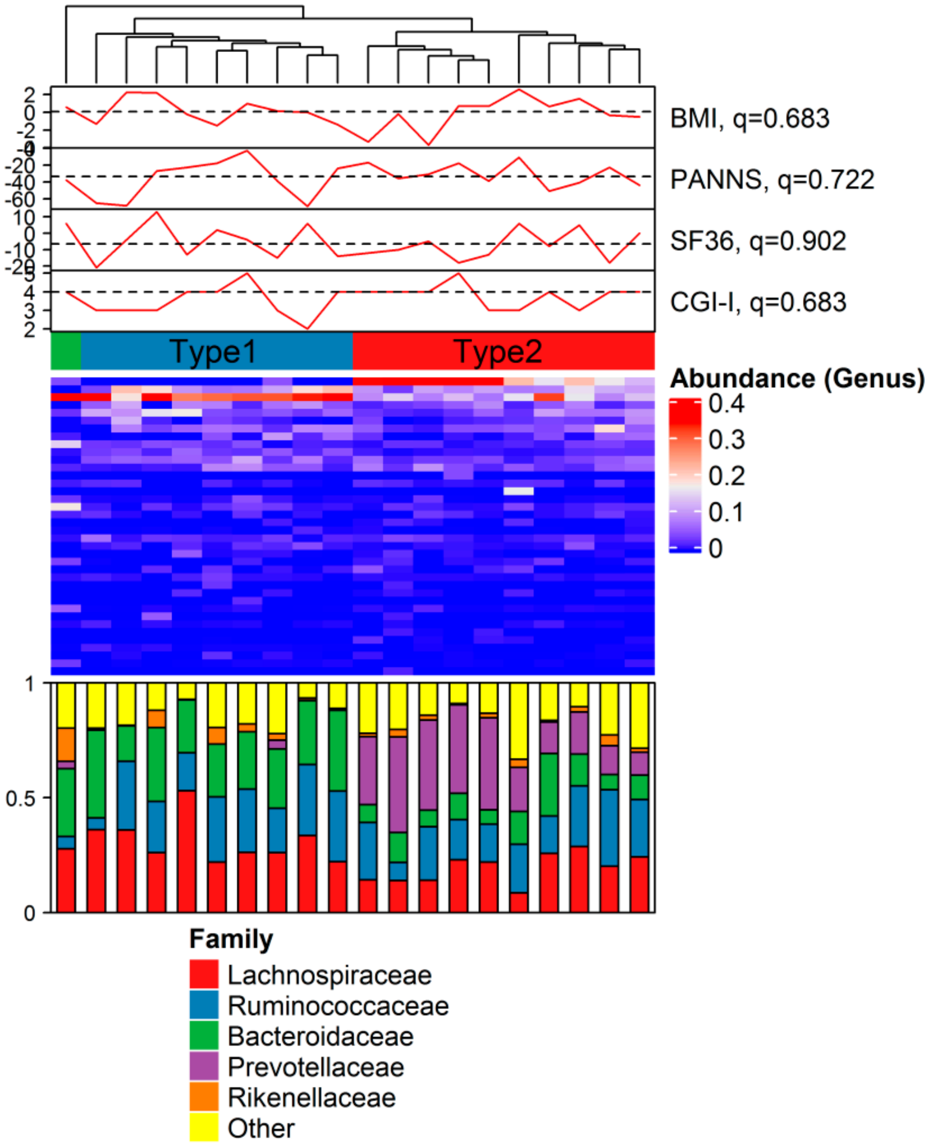

Supplementary Figure S14. Genus level resolution heatmap of relative abundances (in addition to filtering described in methods, OTUs with maximal abundances < 1% were removed). Bottom annotation: Stacked bar plot of relative abundances at the family level (5 most abundant families were included, and the remaining families were combined). Top annotations: PANNS and SD36 changes from baseline; CGI-I at endpoint (W6); horizontal dashed lines in top annotations represent medians; p values, Wilcoxon rank-sum test (Type1 vs. Type2); q, FDR adjusted p values.

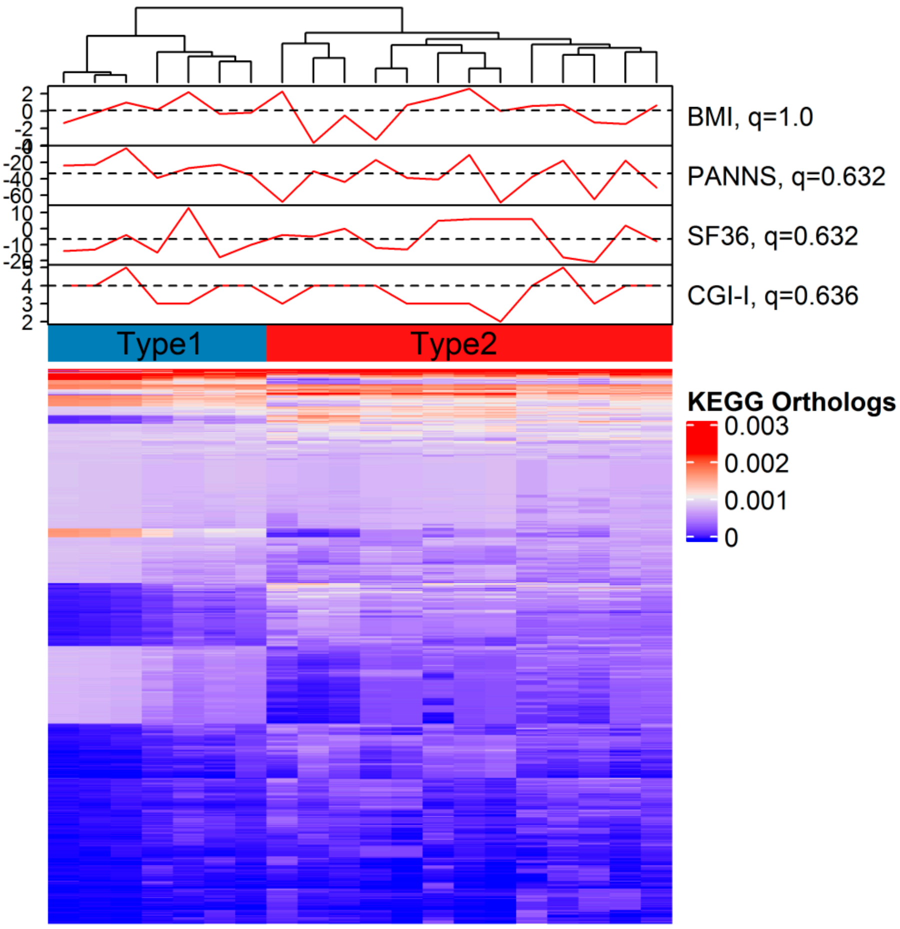

Supplementary Figure S15. Heatmap of relative abundances of the KEGG Orthologs with unsupervised average linkage hierarchical clustering. Features with the maximal abundance of < 0.02% were removed. Top annotations: BMI, PANNS, and SF36 changes from baseline-CGI-I at endpoint (W6); horizontal dashed lines in top annotations represent medians; p values, Wilcoxon rank-sum test (Type1 vs. Type2); q, FDR adjusted p values.

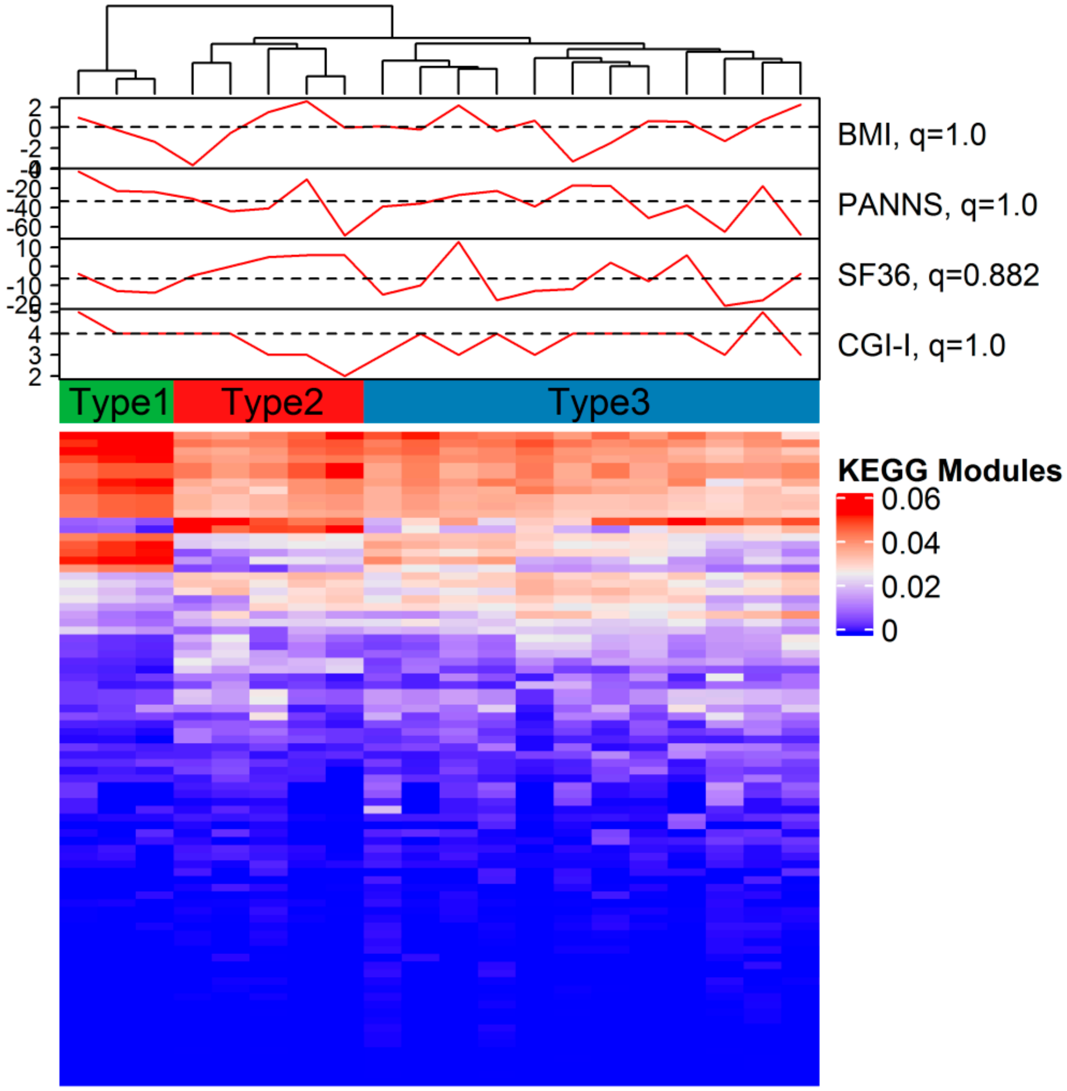

Supplementary Figure S16. Heatmap of relative abundances of the KEGG Modules with unsupervised average linkage hierarchical clustering. Top annotations: BMI, PANNS, and SF36 changes from baseline-CGI-I at endpoint (W6); horizontal dashed lines in top annotations represent medians; p values, Wilcoxon rank-sum test (Type2 vs. Type3); q, FDR adjusted p values.

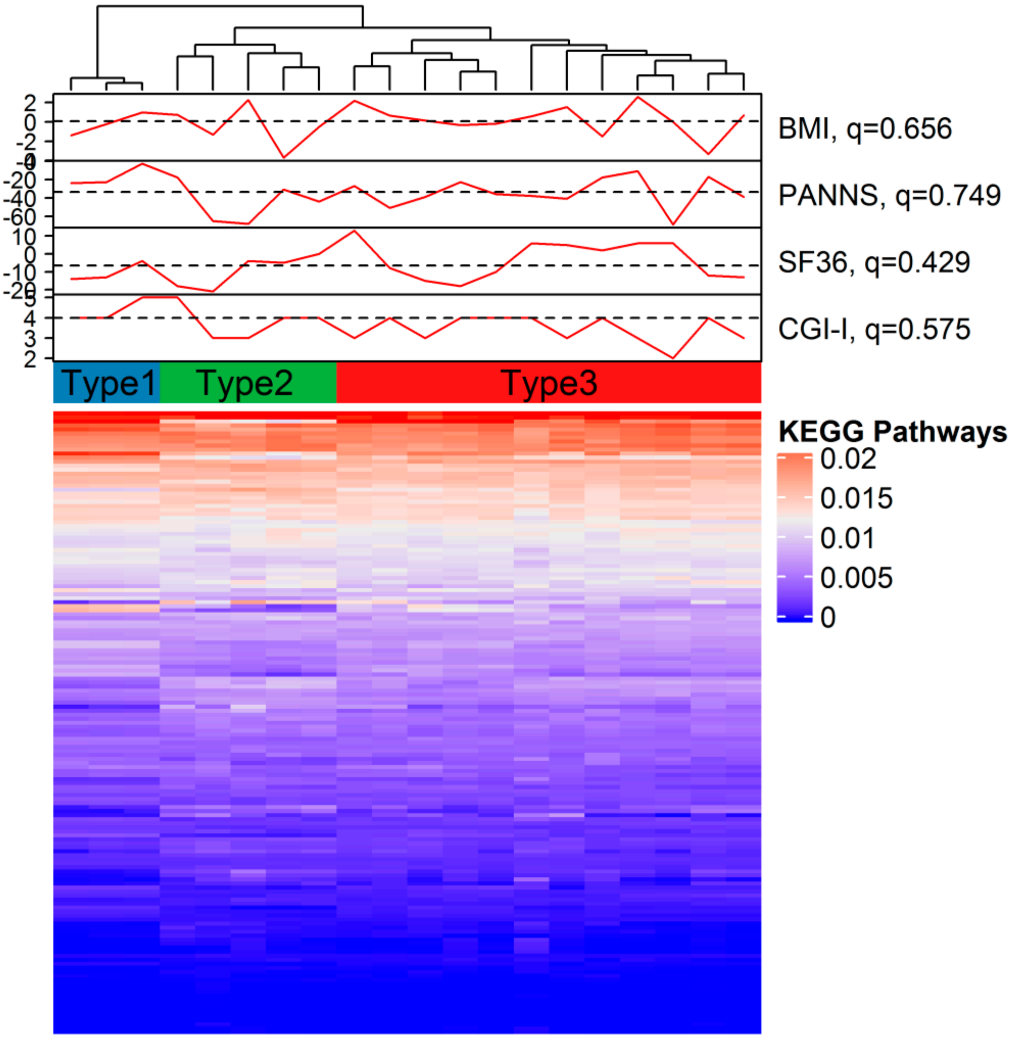

Supplementary Figure S17. Heatmap of relative abundances of the KEGG Pathways with unsupervised average linkage hierarchical clustering. Top annotations: BMI, PANNS, and SF36 changes from baseline-CGI-I at endpoint (W6); horizontal dashed lines in top annotations represent medians; p values, Wilcoxon rank-sum test (Type2 vs. Type3); q, FDR adjusted p values.

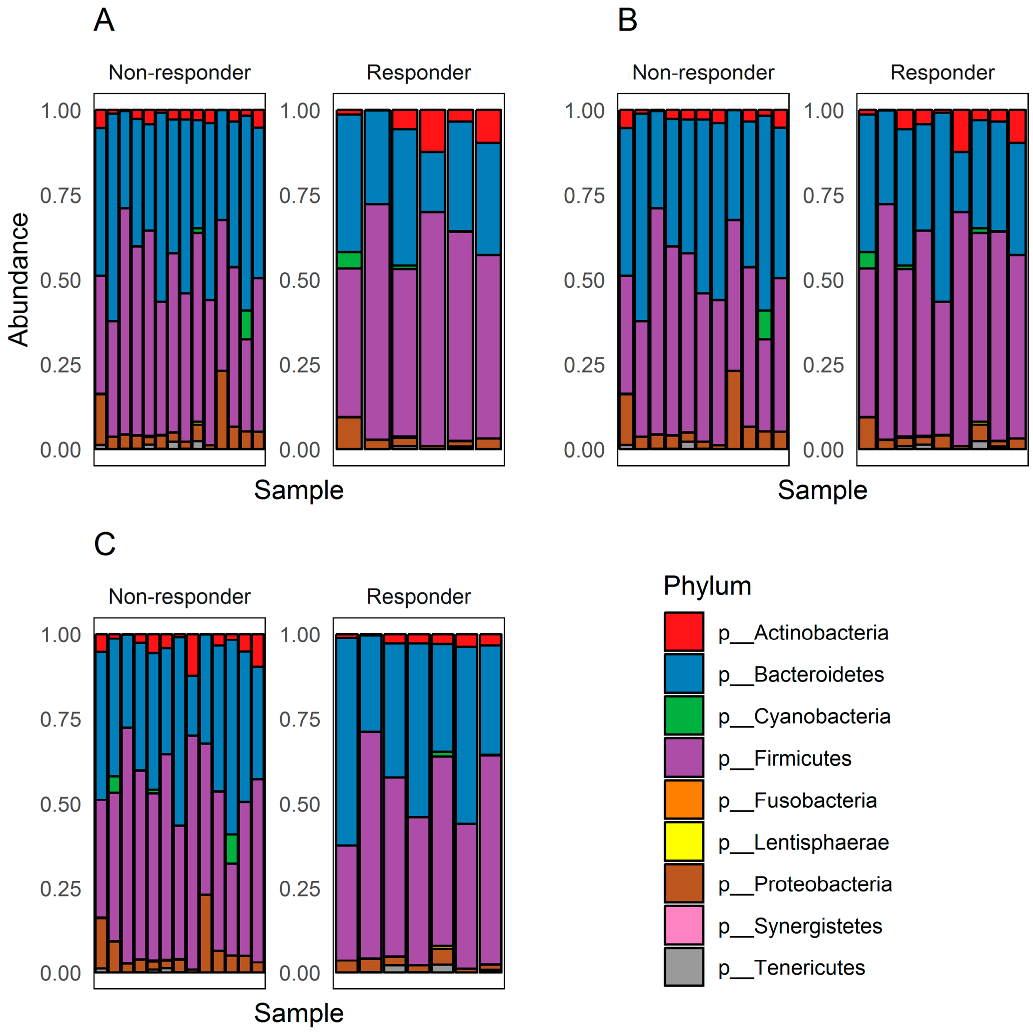

Supplementary Figure S18. Stacked bar plots of phylogenic compositions of bacterial taxa (prevalence 5%) at the phylum level after Olanzapine treatment. Olanzapine treatment classified as (A) Eary responders (6 [30%]) vs Early non-responders (14 [70%]), (B) Late responders (9 [45%]) vs Late non-responders (11 [55%]), and (C) CGI-I responders vs non-responders. Early responders: 30% reduction in PANNS total score at 4 weeks; Late responders: 40% reduction in PANNS total score at end-point; CGI-I responders (7 [35%] score 3- much improvement) vs CGI-I non-responders (13 [65%] score 4 – minimal improvement or 5 – no improvement). Nine most frequent phyla are presented.

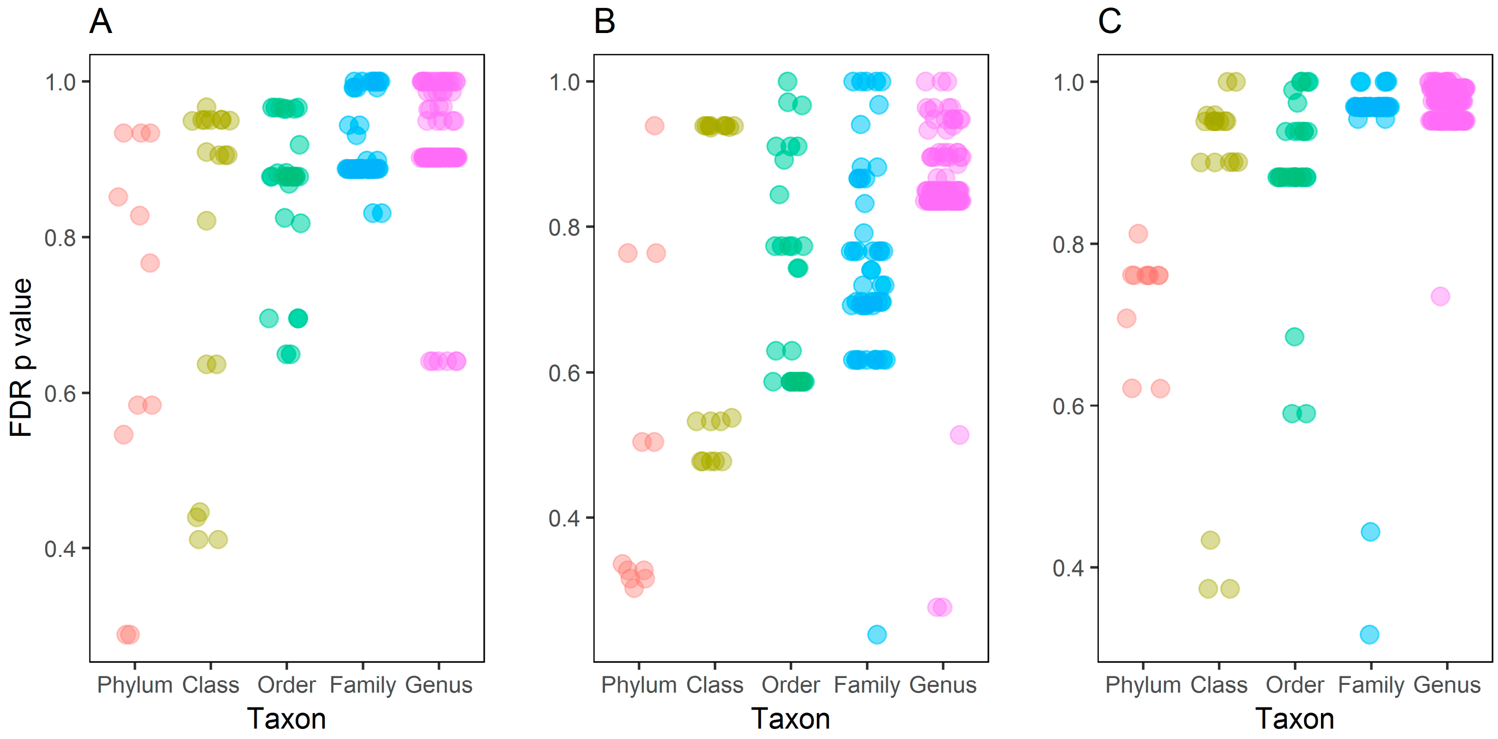

Supplementary Figure S19. Phylogenic compositions - differential abundance testing between responders and non-responders using different definitions of clinical improvements (baseline samples). FDR adjusted p values from differential abundance testing at the phylum, class, order, family, and genus levels using Wilcoxon signed-rank test. (A) Early responders vs. Early non-responders, (B) Late responders vs. Late non-responders, (C) CGI-I responders (CGI-I score 3) vs. non-CGI-I responders (CGI-I score 4 or 5).

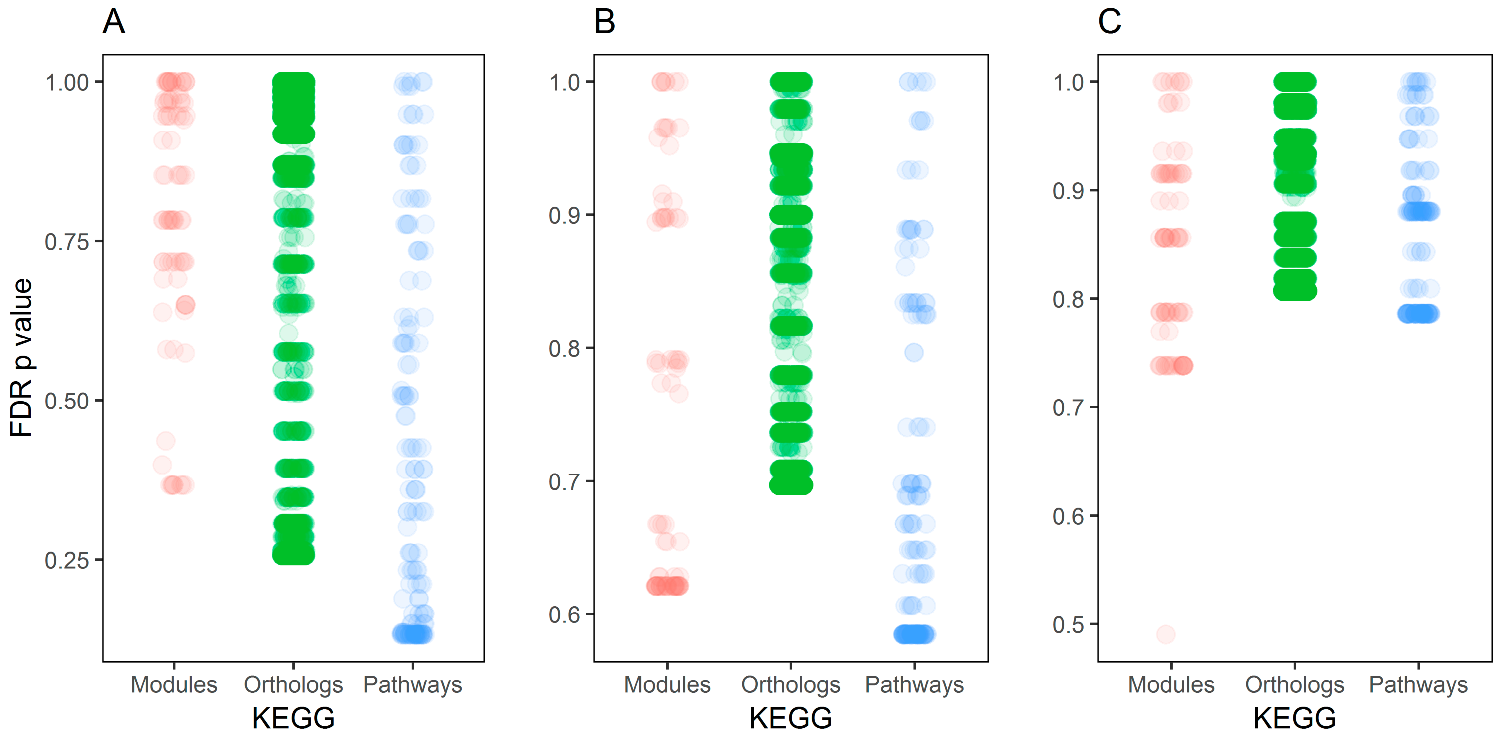

Supplementary Figure S20. KEGG features - differential abundance testing between responders and non-responders using different definitions of clinical improvements (baseline samples). FDR adjusted p values from differential abundance testing of the KEGG Orthologs, Modules, and Pathways using Wilcoxon signed-rank test. (A) Early responders vs. Early non-responders, (B) Late responders vs. Late non-responders, (C) CGI-I responders (CGI-I score 3) vs. non-CGI-I responders (CGI-I score 4 or 5). KEGG Orthologs, Modules, and Pathways abundances were calculated from 16S rRNA sequencing data using PICRUSt and HUManN.

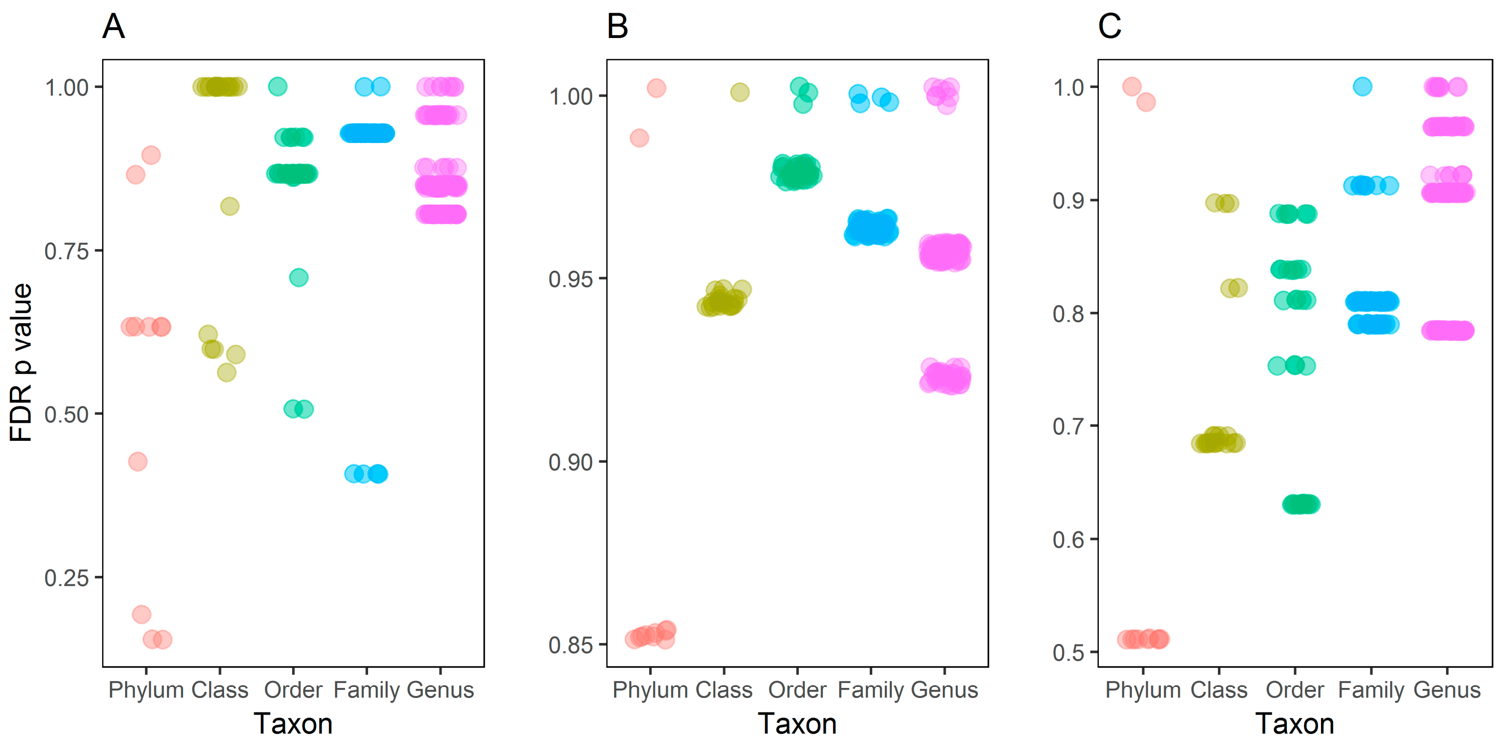

Supplementary Figure S21. Phylogenic compositions- differential abundance testing between responders and non-responders using different definitions of clinical improvements (baseline samples) in females. FDR adjusted p values from differential abundance testing at the phylum, class, order, family, and genus levels using Wilcoxon signed-rank test. (A) Early responders vs. Early non-responders, (B) Late responders vs. Late non-responders, (C) CGI-I responders (CGI-I score 3) vs. non-CGI-I responders (CGI-I score 4 or 5).

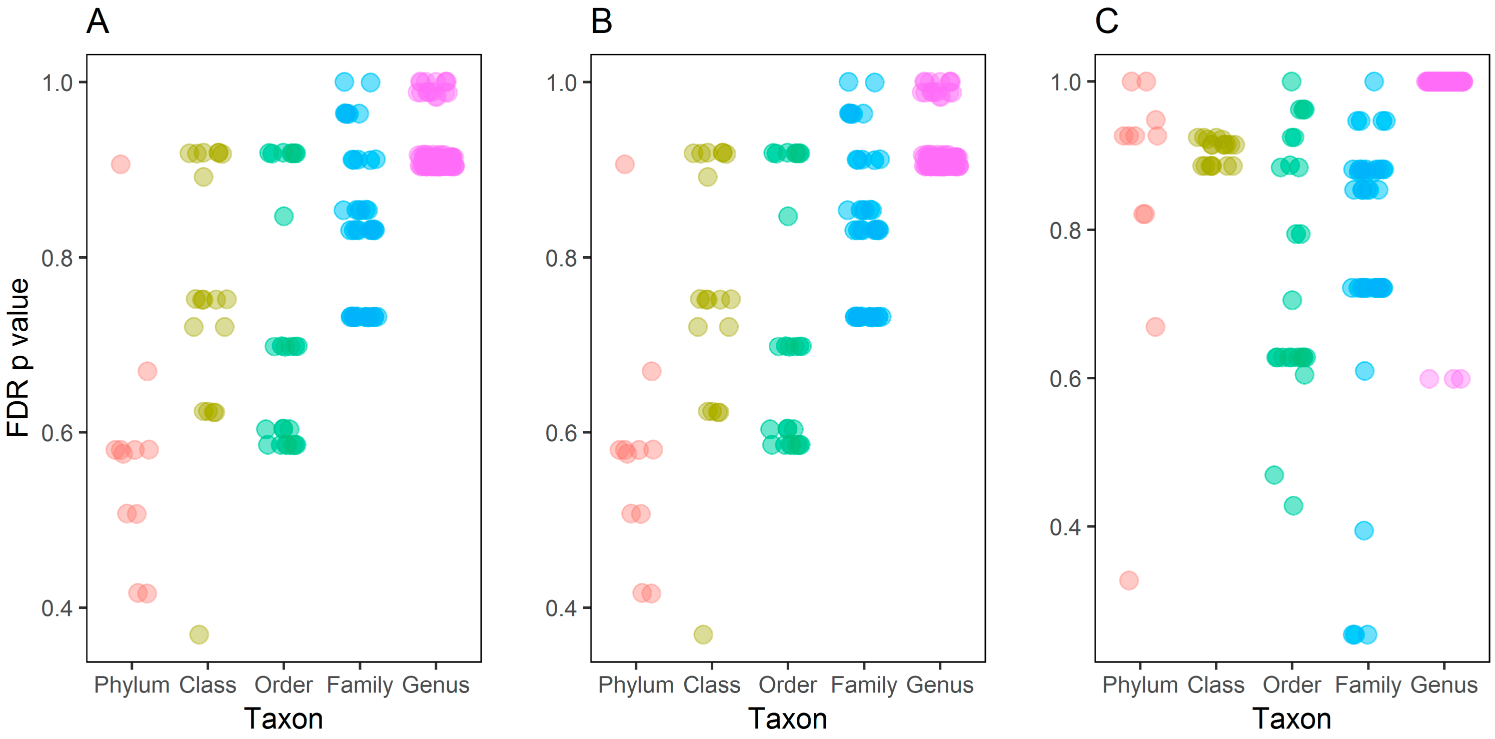

Supplementary Figure S22. Phylogenic compositions - differential abundance testing between responders and non-responders using different definitions of clinical improvements (baseline samples) in males. FDR adjusted p values from differential abundance testing at the phylum, class, order, family, and genus levels using the Wilcoxon signed-rank test. (A) Early responders vs. Early non-responders, (B) Late responders vs. Late non-responders, (C) CGI-I responders (CGI-I score 3) vs. non-CGI-I responders (CGI-I score 4 or 5).

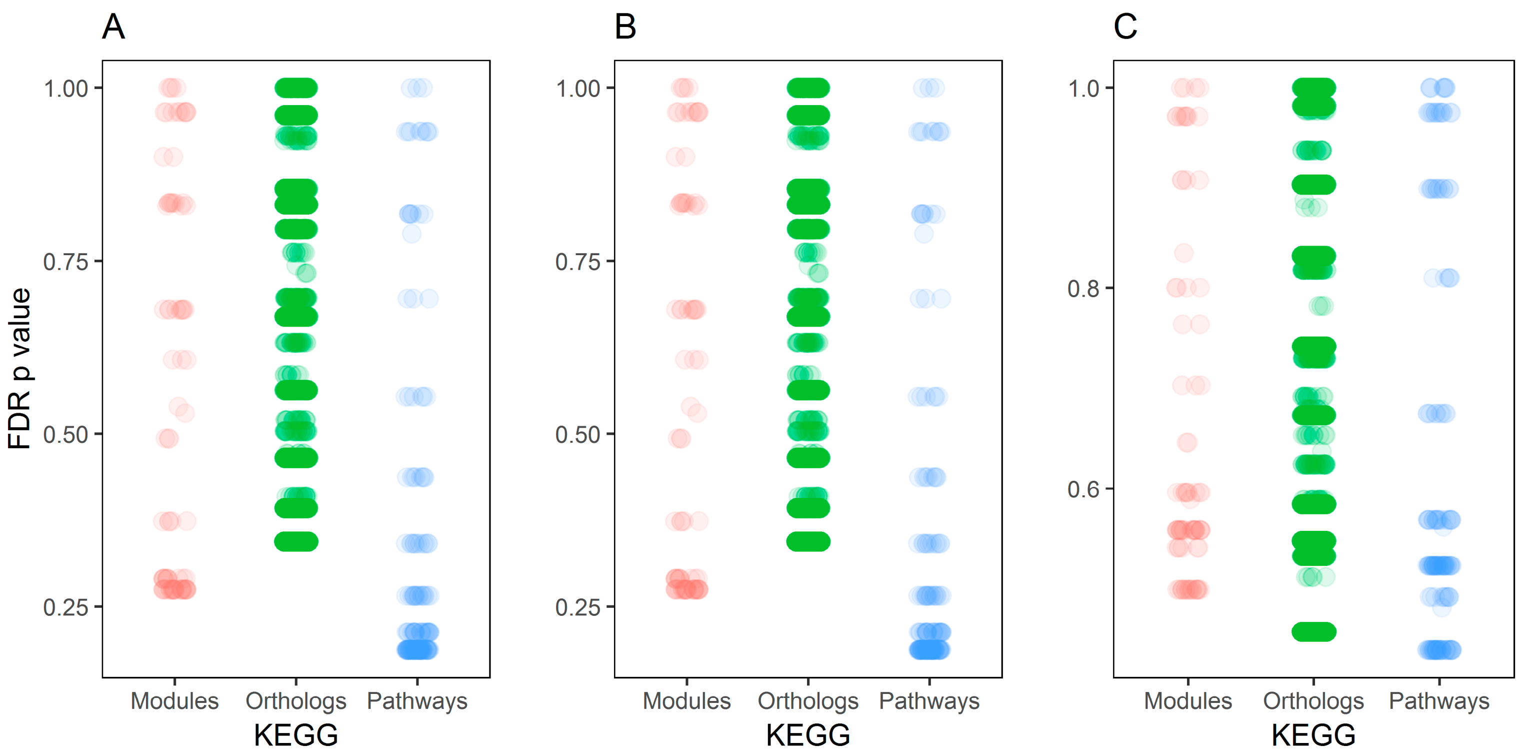

Supplementary Figure S23. KEGG features - differential abundance testing between responders and non-responders using different definitions of clinical improvements (baseline samples) in males. FDR adjusted p values from differential abundance testing of the KEGG Orthologs, Modules, and Pathways using Wilcoxon signed-rank test. (A) Early responders vs. Early non-responders, (B) Late responders vs. Late non-responders, (C) CGI-I responders (CGI-I score 3) vs. non-CGI-I responders (CGI-I score 4 or 5). KEGG Orthologs, Modules, and Pathways abundances were calculated from 16S rRNA sequencing data using PICRUSt and HUManN.

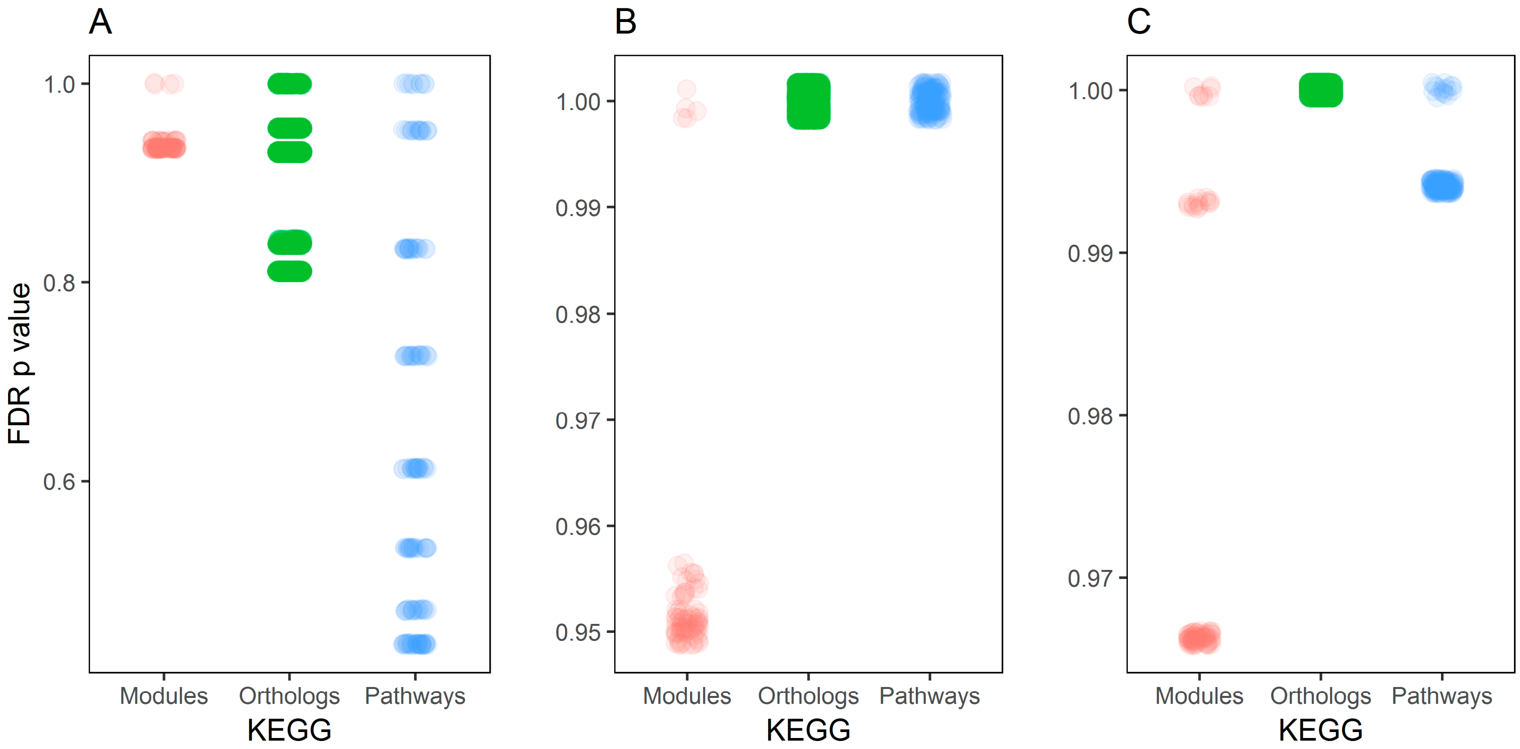

Supplementary Figure S24. KEGG features - differential abundance testing between responders and non-responders using different definitions of clinical improvements (baseline samples) in females. FDR adjusted p values from differential abundance testing of the KEGG Orthologs, Modules, and Pathways using Wilcoxon signed-rank test. (A) Early responders vs. Early non-responders, (B) Late responders vs. Late non-responders, (C) CGI-I responders (CGI-I score 3) vs. non-CGI-I responders (CGI-I score 4 or 5). KEGG Orthologs, Modules, and Pathways abundances were calculated from 16S rRNA sequencing data using PICRUSt and HUManN.

Supplementary references

1. Jarosz, M.; Rychlik, E.; Stoś, K.; Wierzejska, R.; Wojtasik, A.; Charzewska, J.; Mojska, H.; Szponar, L.; Sajór, I.; Kłosiewicz-Latoszek, L.; et al. *Normy żywienia dla populacji Polski*; Instytut Żywności i Żywienia: Warszawa, 2017; ISBN 978-83-86060-89-4.
2. Arumugam, M.; Raes, J.; Pelletier, E.; Le Paslier, D.; Yamada, T.; Mende, D.R.; Fernandes, G.R.; Tap, J.; Bruls, T.; Batto, J.-M.; et al. Enterotypes of the human gut microbiome. *Nature* **2011**, *473*, 174–180.
